# HAND2 is a novel obesity-linked adipogenic transcription factor regulated by glucocorticoid signalling

**DOI:** 10.1007/s00125-021-05470-y

**Published:** 2021-05-20

**Authors:** Maude Giroud, Foivos-Filippos Tsokanos, Giorgio Caratti, Stefan Kotschi, Sajjad Khani, Céline Jouffe, Elena S. Vogl, Martin Irmler, Christina Glantschnig, Manuel Gil-Lozano, Daniela Hass, Asrar Ali Khan, Marcos Rios Garcia, Frits Mattijssen, Adriano Maida, Daniel Tews, Pamela Fischer-Posovszky, Annette Feuchtinger, Kirsi A. Virtanen, Johannes Beckers, Martin Wabitsch, Henriette Uhlenhaut, Matthias Blüher, Jan Tuckermann, Marcel Scheideler, Alexander Bartelt, Stephan Herzig

**Affiliations:** 1 Institute for Diabetes and Cancer (IDC); Helmholtz Zentrum München, German Research Center for Environmental Health, Neuherberg, Germany; 2 German Center for Diabetes Research (DZD), Neuherberg, Germany; 3 Joint Heidelberg-IDC Translational Diabetes Program, Inner Medicine 1, Heidelberg University Hospital, Heidelberg, Germany; 4 Institute for Cardiovascular Prevention (IPEK), Ludwig-Maximilians-University, Munich, Germany; 5 Institute for Comparative Molecular Endocrinology, Universität Ulm, Ulm, Germany; 6 Institute of Experimental Genetics, Helmholtz Zentrum München, Neuherberg, Germany; 7 Division of Pediatric Endocrinology and Diabetes, Department of Pediatrics and Adolescent Medicine, Ulm University Medical Center, Ulm, Germany; 8 Research Unit Analytical Pathology, Helmholtz Center Munich, Neuherberg, Germany; 9 Turku PET Centre, Turku University Hospital, Turku, Finland; 10 Experimental Genetics, TUM School of Life Sciences, Technische Universität München, Freising, Germany; 11 Metabolic Programming, TUM School of Life Sciences Weihenstephan and ZIEL Institute for Food & Health, Munich, Germany; 12 Helmholtz Institute for Metabolic, Obesity and Vascular Research (HI-MAG) of the Helmholtz Zentrum München at the University of Leipzig and University Hospital Leipzig, Leipzig, Germany; 13 German Center for Cardiovascular Research (DZHK), Partner Site Munich Heart Alliance, Munich, Germany; 14 Department of Molecular Metabolism, Harvard T.H. Chan School of Public Health, Boston, MA USA; 15 Molecular Metabolic Control, Medical Faculty, Technical University Munich, Munich, Germany

**Keywords:** Adipocytes, Dexamethasone, Differentiation, Glucocorticoid receptor, HAND2, hMADS, Human adipose tissue, Mesenchymal stem cells, Obesity, Transcription factor

## Abstract

**Aims/hypothesis:**

Adipocytes are critical cornerstones of energy metabolism. While obesity-induced adipocyte dysfunction is associated with insulin resistance and systemic metabolic disturbances, adipogenesis, the formation of new adipocytes and healthy adipose tissue expansion are associated with metabolic benefits. Understanding the molecular mechanisms governing adipogenesis is of great clinical potential to efficiently restore metabolic health in obesity. Here we investigate the role of heart and neural crest derivatives-expressed 2 (HAND2) in adipogenesis.

**Methods:**

Human white adipose tissue (WAT) was collected from two cross-sectional studies of 318 and 96 individuals. In vitro, for mechanistic experiments we used primary adipocytes from humans and mice as well as human multipotent adipose-derived stem (hMADS) cells. Gene silencing was performed using siRNA or genetic inactivation in primary adipocytes from loxP and or tamoxifen-inducible Cre-ERT2 mouse models with Cre-encoding mRNA or tamoxifen, respectively. Adipogenesis and adipocyte metabolism were measured by Oil Red O staining, quantitative PCR (qPCR), microarray, glucose uptake assay, western blot and lipolysis assay. A combinatorial RNA sequencing (RNAseq) and ChIP qPCR approach was used to identify target genes regulated by HAND2. In vivo, we created a conditional adipocyte *Hand2* deletion mouse model using Cre under control of the *Adipoq* promoter (*Hand2*^AdipoqCre^) and performed a large panel of metabolic tests.

**Results:**

We found that HAND2 is an obesity-linked white adipocyte transcription factor regulated by glucocorticoids that was necessary but insufficient for adipocyte differentiation in vitro. In a large cohort of humans, WAT *HAND2* expression was correlated to BMI. The *HAND2* gene was enriched in white adipocytes compared with brown, induced early in differentiation and responded to dexamethasone (DEX), a typical glucocorticoid receptor (GR, encoded by *NR3C1*) agonist. Silencing of *NR3C1* in hMADS cells or deletion of GR in a transgenic conditional mouse model results in diminished *HAND2* expression, establishing that adipocyte HAND2 is regulated by glucocorticoids via GR in vitro and in vivo. Furthermore, we identified gene clusters indirectly regulated by the GR–HAND2 pathway. Interestingly, silencing of *HAND2* impaired adipocyte differentiation in hMADS and primary mouse adipocytes. However, a conditional adipocyte *Hand2* deletion mouse model using Cre under control of the *Adipoq* promoter did not mirror these effects on adipose tissue differentiation, indicating that HAND2 was required at stages prior to *Adipoq* expression.

**Conclusions/interpretation:**

In summary, our study identifies HAND2 as a novel obesity-linked adipocyte transcription factor, highlighting new mechanisms of GR-dependent adipogenesis in humans and mice.

**Data availability:**

Array data have been submitted to the GEO database at NCBI (GSE148699).

**Graphical abstract:**

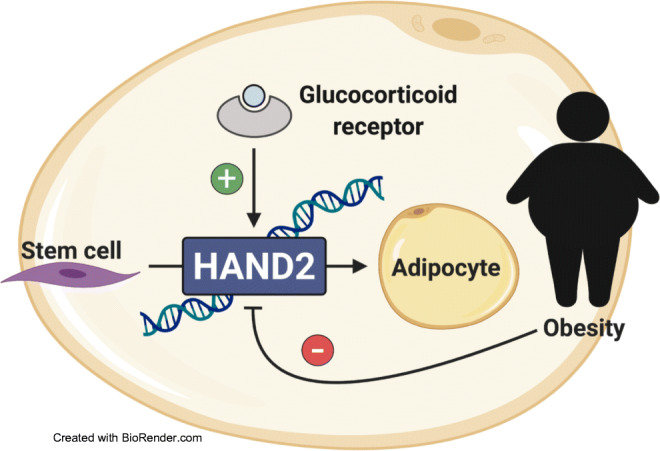

**Supplementary Information:**

The online version contains peer-reviewed but unedited supplementary material available at 10.1007/s00125-021-05470-y.

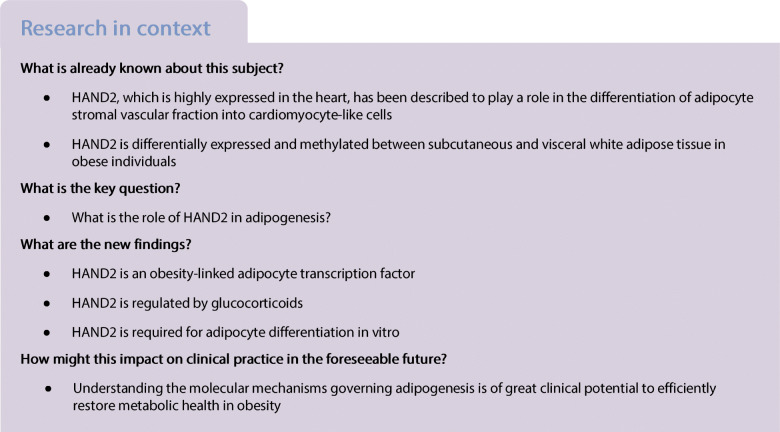



## Introduction

Adipocytes are specialised cells that have a variety of functions including nutrient buffering, endocrine regulation and thermogenesis. Principally, there are two types of adipocytes: first, white adipocytes, able to store and release lipids and secrete adipokines and, second, thermogenic adipocytes (brown and beige), which additionally dissipate chemical energy from nutrients as heat [1]. Obesity, the excess accumulation of white adipose tissue (WAT), is characterised by adipocyte dysfunction and metabolic imbalance. Enhancing adipocyte health or increasing the number of functional adipocytes has beneficial effects on systemic metabolism.

Adipogenesis is a complex process regulated by the interplay of transcription factors, metabolites and hormonal cues. The commitment of mesenchymal stem cells to becoming pre-adipocytes and adipogenesis occur in waves of transcriptional programs. Genes involved in development, for example those encoding bone morphogenic proteins, Wnt or hedgehog proteins, play a critical role during the commitment of progenitor cells to the adipocyte lineage [2] while activating peroxisome proliferator-activated receptor γ (PPARγ), for example by rosiglitazone, promotes later adipocytes differentiation and induces a thermogenic phenotype [3].

Glucocorticoids (GCs) are steroid hormones modulating metabolism [4–6] including adipogenesis, lipolysis, lipogenesis and thermogenesis [7]. However, recent studies have demonstrated that the glucocorticoid receptor (GR) is not required for the development of adipose tissue in mouse models [8–11]. Nevertheless, the effects of GCs on adipocytes are multifaceted and largely depend on the metabolic status of the individual. Chronically elevated levels of GCs, either by pharmacological treatment or in patients with Cushing’s syndrome, results in partial lipodystrophy [12]. However, neither the adipose-specific activation of GR by GCs nor their regulation in obesity are fully understood.

In search of novel adipose-specific mechanisms linked to human obesity we have previously investigated methylation and gene expression signatures of visceral WAT (visWAT) and subcutaneous WAT (scWAT) and found that the transcription factor HAND2 (heart and neural crest derivatives-expressed 2) is among the most differentially expressed genes [13]. *HAND2* encodes a helix-loop-helix transcription factor that plays a role in cardiac morphogenesis by binding other helix-loop-helix proteins including its close relative HAND1 [14]. This ability of HAND2 to control organ development [15–18] and cell differentiation led us to hypothesise that HAND2 might be involved in adipocyte differentiation and function.

## Methods

For detailed methods, please refer to the Electronic supplementary material (ESM).

### Cell culture and stromal vascular fraction preparation

Human multipotent adipose-derived stem (hMADS) cells were kindly provided by E.Z. Amri. hMADS were free of mycoplasma contamination and cultivated as previously described [19, 20]. Mouse stromal vascular fraction (SVF; mSVF) was isolated from brown adipose tissue (BAT), scWAT, and visWAT, and differentiated into adipocytes [21]. Human SVF (hSVF) was isolated from scWAT, collected from healthy patients (abdominoplasty) and differentiated by administering the same adipogenic cocktail as for hMADS [21]. The study was approved by the University of Ulm Ethics Committee (vote no. 300/16) and all donors gave written informed consent.

### Gene expression and functional analysis in vitro

HAND2 and NR3C1 mRNA was silenced in hSVF, hMADS and mSVF using 20 nmol/l siRNA (SMARTpool ON-TARGETplus siRNA Horizon Discovery, UK), cells were collected 48 h after transfection. To overexpress Hand2 we infected mSVF or 3T3-L1 cells with the pcDNA-3XFlag-*Hand2* vector [15] and the pENTR-CMV-MCS-TKpA as control using TransIT-X2 Dynamic Delivery System (Mirus, USA, MIR 6003). Cells were collected 72 h after infection. Chemical activation and inhibition of GR were performed by 12 h treatment with respectively 1 μmol/l dexamethasone (DEX) (Merck, Germany D4902) and/or 2 μmol/l RU486 (475838, Merck). Transcriptional analysis was performed using SYBR-based quantitative PCR (qPCR) (primers listed in ESM Table 1), microarray (Human Clariom S arrays, Thermo Fisher Scientific, Germany) and RNAseq (performed by Novogene, UK). Protein levels of Akt, pAkt and β-actin (Cell Signaling, Germany #9272, #4051, #4970, respectively, 1/1000) were quantified by western blot [22]. Oil Red O staining, glycerol accumulation and glucose uptake was measured. ChIP qPCR was performed as previously described [23].

### Mouse experiments

All animal studies were conducted in accordance with German animal welfare legislation and protocols were approved by the state Ethics Committee and Government of Upper Bavaria (nos. ROB-55.2-2532.Vet_02-16-117; ROB-55.2-2532.Vet_02-17-125; ROB-55.2-2532.Vet_02-15-164). All mice were group-housed and maintained in a climate-controlled environment at 22°C with a 12 h dark–light cycle under specific pathogen-free conditions in the animal facility of the Helmholtz Center Munich. *db/db* mice (JAX mouse strain) were purchased from Charles River (https://www.criver.com/products-services/find-model/jax-dbdb-mice?region=23) Adipocyte-specific *Hand2* knockout mice (*Hand2*^AdipoqCre^) were generated by crossing *Adipoq*^CRE^ mice (Jackson laboratory, stock number 028020; C57BL/6J, https://www.jax.org/strain/028020) with *Hand2*^flox/flox^ mice (NMRI strain), kindly provided by R. Zeller [24]. *Hand2*^AdipoqCre^ (CRE+) and wild-type littermates (CRE−) were used for all experiments. Animals were fed a high-fat diet (HFD) 60% energy from fat (Research Diets, New Brunswick, NJ, USA D12492) ad libitum from the age of 6 weeks for 12 weeks, after which glucose and insulin tolerance tests were performed. Briefly, animals were fasted and subsequently injected intraperitoneally with glucose at 2 g/kg or insulin 0.8 U/kg. Body mass composition measurement, necropsy, and haematoxylin/eosin (HE) staining were performed. Histological staining with HE was performed on 3 μm thick sections of BAT, scWAT and gWAT. Intraperitoneal injection of DEX (1 mg/kg) (Sigma, Germany #D9184-5G) was performed on wild-type C57BL/6J (JAX mouse strain) (Charles River, https://www.criver.com/products-services/find-model/jax-c57bl6j-mice) for 6 h, 2 days or 2 weeks and *Hand2*^AdipoqCre^ for 6 h. Indirect calorimetry, including energy expenditure, food consumption, oxygen consumption and locomotor activity, was measured for *Hand2*^AdipoqCre^ using metabolic cages (TSE PhenoMaster cages TSE Systems, Bad Homburg, Germany). *GR*^flox/flox^ and *GR*^ERT2Cre^ (on a C57BL/6 background) and *Hand2*^3XFlag^ (on an NMRI background) mice were generated as previously described [15, 25]. *Hand2* expression was analysed using SYBR-based qPCR (Thermo Fisher, Germany #A25741) (primers listed in ESM Table 1).

### Human studies

The study protocol relating to human BAT was approved by the Ethics Committee of the Hospital District of Southwestern Finland, and participants provided written informed consent following the committee's instructions. The study was conducted according to the principles of the Declaration of Helsinki. Human BAT was collected from fluorodeoxyglucose F18-positron emission tomography-positive scan areas in the supraclavicular location and scWAT was taken from the same incision. The studies referring to the human cohort 1 and 2 were approved by the Ethics Committee of the University of Leipzig (approval no: 159-12-21052012) and performed in accordance to the declaration of Helsinki. All participants gave written informed consent before taking part in the study*.* Human cohort 1 refers to human scWAT versus visWAT samples collected in the context of a cross-sectional study of 318 individuals (249 women, 69 men; BMI range: 21.9–97.3 kg/m^2^, age range: 19–75 years). An additional 13 individuals receiving DEX treatment for chronic inflammatory diseases were compared to BMI-matched (35–67 kg/m^2^) individuals from cohort 1. In cohort 2, 96 individuals were selected from the Leipzig Obesity Biobank to define age- and sex-matched groups of healthy lean individuals (*n* = 32; mean BMI: 23.4 ± 1.5 kg/m^2^ mean age 43.6 ± 7.1 years, 23 female, 9 male participants), individuals with metabolically healthy obesity (*n* = 32; mean BMI: 45.9 ± 6.8 kg/m^2^ mean age 42.6 ± 9.3 years, 23 female, 9 male participants) further BMI-matched to 32 individuals with obesity and type 2 diabetes (mean BMI: 45.3 ± 4.7 kg/m^2^ mean age 42.7 ± 6.7 years, 25 female, 7 male participants).

*HAND2* mRNA expression was measured using SYBR-based qPCR (primers listed in ESM Table 1).

### Statistics

Data presented as bar charts were expressed as mean ± SEM. Data presented as box and whisker plots were shown as median with upper and lower quartile ± maximum and minimum points. Two-tailed, unpaired *t* test was used when comparing two conditions. One-way ANOVA and two-way ANOVA with Tukey test were used when comparing three or more groups, as reported in the figure legends. Analysis was performed using GraphPad Prism. A *p* value <0.05 was considered significant as indicated by asterisks in the figure legends. Mouse experiments involving *Hand2*^AdipoqCre^ were performed three times independently and pooled. Mice were excluded for poor body condition or if they died before the end of the experiment. For the quantification of *HAND2* in control patients versus DEX-treated patients, only patients in the same range of BMI were considered, and outliers were excluded using the ROUT test Q1% from GraphPad Prism.

## Results

### Adipose *HAND2* is correlated to obesity in mice and humans

**Fig. 1:**
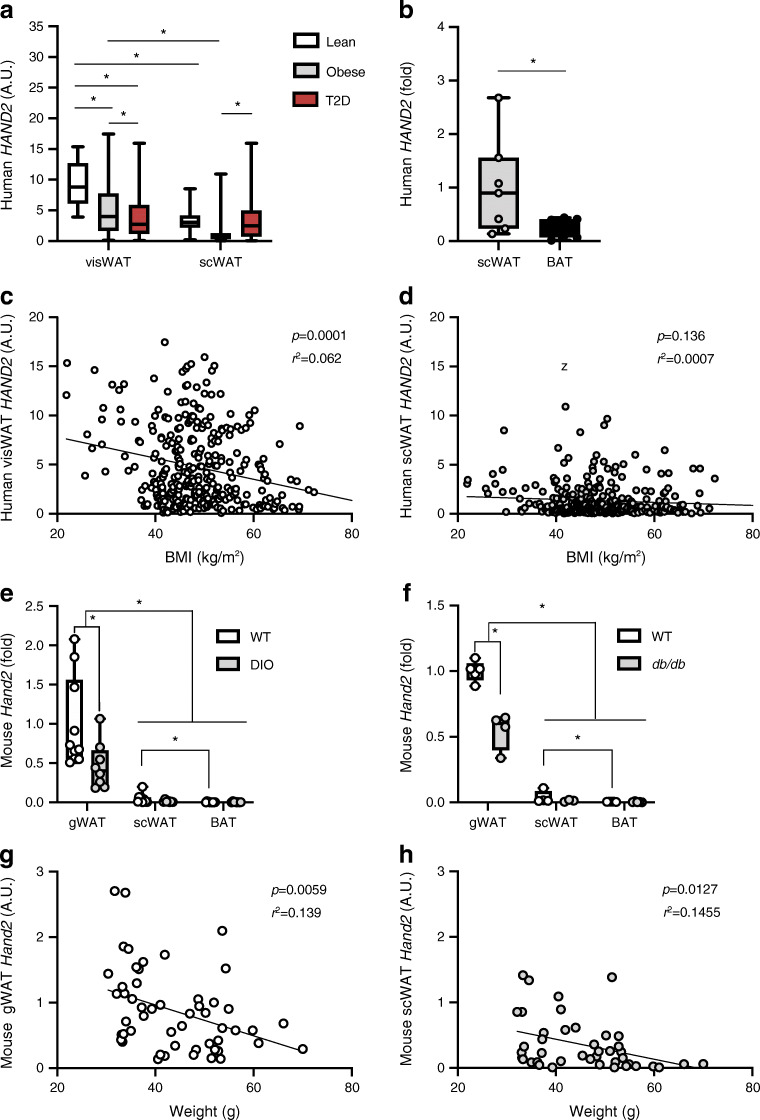
White adipocyte *HAND2* is correlated to obesity in mice and humans. (**a**, **b**) *HAND2* expression in visWAT vs scWAT from lean, obese or diabetic participants (*n* = 318 participants; cohort 1) (**a**), and in human scWAT vs BAT (*n* = 7 individuals) (**b**). (**c**, **d**) *HAND2* expression correlated with BMI in visWAT (**c**) and scWAT (**d**) (*n* = 318 participants; cohort 1). (**e**, **f**) *Hand2* expression in wild-type vs DIO mice (*n* = 10 mice) (**e**) and in WT vs *db/db* mice (*n* = 5 mice) (**f**). (**g**, **h**) *Hand2* expression correlated with body weight in mouse gWAT (**g**) and scWAT (**h**) (*n* = 54 mice). Data are presented as arbitrary units representing copy number of *HAND2* normalised to *HPRT1* (**a**, **c**, **d**) or *Tbp* (**g**, **h**); as fold change compared to scWAT (**b**); or as fold change compared to gWAT/WT (**e**, **f**). Statistics: one-way ANOVA with Tukey test (**a**), two-tailed paired *t* test (**b**), correlation (**c**, **d**, **g**, **h**), two-way ANOVA with Tukey test (**e**, **f**). Data are presented as median with upper and lower quartile ± maximum and minimum (**a, b, e**, **f**). Statistical significance is indicated by *p < 0.05. A.U., arbitrary units; T2D, type 2 diabetes; WT, wild-type

In order to investigate the importance of HAND2 in adipose tissue biology, we first determined its gene expression in different adipose depots obtained from groups of non-obese, obese and diabetic individuals over a broad range of BMI. *HAND2* mRNA levels were higher in visWAT compared with scWAT (cohort 1) (ESM Fig. 1a) and were lower in obese or diabetic participants compared with lean participants, especially in the visWAT (cohort 1) (Fig. 1a). We confirmed these findings in a second independent cohort (cohort 2) consisting of an equal number of lean, obese and diabetic participants (ESM Fig. 1b). ScWAT depots showed substantially higher *HAND2* expression than human BAT (Fig. 1b). Intriguingly, *HAND2* expression in visWAT but not in scWAT was inversely correlated with BMI in these patients (Fig. 1c,d). However, *HAND2* in both tissues was correlated with body weight in both cohorts (ESM Table 2). In line with these human data, in mice *Hand2* expression was, as expected, highest in the heart followed by liver and gonadal WAT (gWAT), with lower expression in scWAT, BAT and gastrocnemius muscle (ESM Fig. 1c). Moreover, gWAT *Hand2* was significantly lower in mouse models of obesity, including HFD-induced obesity (referred to here as diet-induced obesity [DIO]) as well as genetically obese, leptin receptor-deficient *db*/*db* mice (Fig. 1e,f), and also inversely correlated with body weight (Fig. 1g,h). These data demonstrate that HAND2 was prominently expressed in gWAT and correlated with obesity in both mice and humans.

### *HAND2* is expressed in adipocytes and is induced early in adipogenesis

**Fig. 2:**
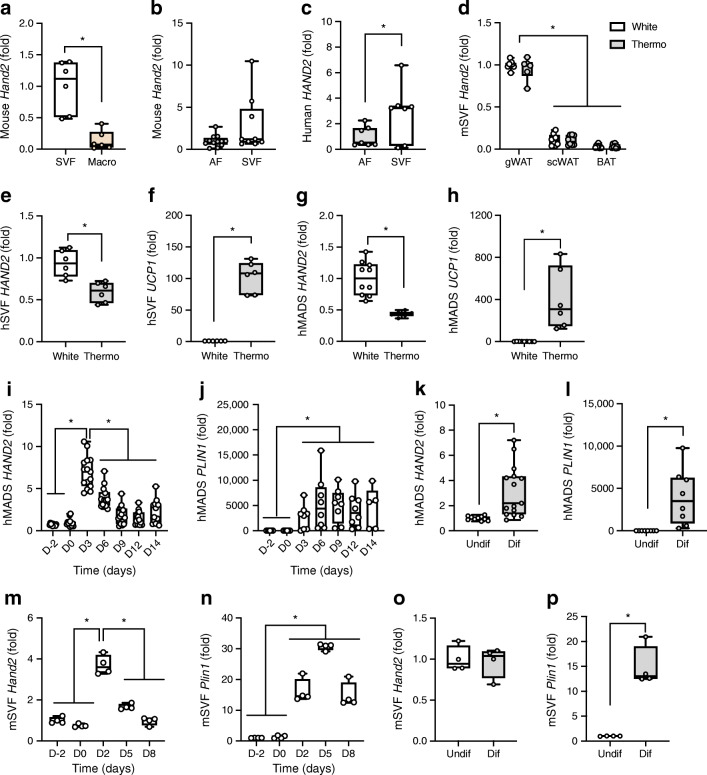
*HAND2* is induced in early differentiation and expressed in both pre-adipocytes and mature adipocytes. (**a**–**c**) *Hand2* expression in mSVF vs macrophages (*n* = 6 replicates) (**a**), vs adipocyte fraction (AF *n* = 12 replicates, SVF *n* = 9 replicates) (**b**) and in human AF vs hSVF (*n *= 7 replicates) (**c**). (**d**) *Hand2* expression in mSVF from different fat depots differentiated using rosiglitazone into white or thermogenic adipocytes (*n* = 8 replicates). (**e**, **f**) *HAND2* (**e**) and *UCP1* (**f**) expression in hSVF differentiated into adipocytes (White *n* = 9 replicates, Thermo *n* = 6 replicates). (**g**, **h**) *HAND2* (**g**) and *UCP1* (**h**) expression in hMADS mature adipocytes (White *n* = 10 replicates, Thermo *n* = 6 replicates). (**i**, **j**) *HAND2* (**i**) and *PLIN1* (**j**) expression in hMADS cells during differentiation (*n* = 9–15 replicates). (**k**, **l**) *HAND2* (**k**) and *PLIN1* (**l**) expression in hMADS pre-adipocytes and mature adipocytes (*n* = 12 replicates). (**m**, **n**) *Hand2* (**m**) and *Plin1* (**n**) expression during mSVF differentiation (*n* = 4 replicates). (**o**, **p**) *Hand2* (**o**) and *Plin1* (**p**) expression in mSVF pre-adipocytes and mature adipocytes (*n* = 4 replicates). Data are presented as fold change compared to the condition mSVF (**a**); to the condition AF (**b, c**); to the condition gWAT/White (**d**); to the condition White (**e–h**); to the condition D-2 (**i**, **j**, **m**, **n**); to the condition Undif (**k**, **l**, **o**, **p**). Statistics: two-tailed unpaired *t* test (**a**, **b**, **c**, **e**, **f**, **g**, **h**, **k**, **l**, **o**, **p**); one-way ANOVA with Tukey test (**i**, **j**, **m**, **n**), two-way ANOVA with Tukey test (**d**); median with upper and lower quartile ± maximum and minimum (**a–p**). Statistical significance is indicated by *p < 0.05. AF, adipocyte fraction; D, day; Dif, differentiated; Macro, macrophages; Thermo, thermogenic; Undif, undifferentiated

The cellular composition of adipose tissue is heterogenous and changes dynamically in obesity. To analyse the origin of HAND2 expression in more detail, we next measured *HAND2* mRNA levels in fractionated adipose tissue from mouse and human samples ex vivo. Mouse *Hand2* was poorly expressed in the macrophage fraction and its levels were not significantly higher in mSVF compared with the adipocyte fraction (*p* = 0.2) (Fig. 2a,b) while in human adipose tissue, *HAND2* levels were higher in the hSVF compared with the adipocyte fraction (Fig. 2c). These findings indicate that HAND2 was predominantly expressed in pre-adipocytes and adipocytes. To further evaluate this concept, we employed in vitro adipocyte models. Differentiated adipocytes derived from the mSVF of distinct anatomical locations mirrored the in vivo *Hand2* mRNA expression pattern with highest levels in adipocytes from gWAT compared with those isolated from scWAT or from BAT (Fig. 2d). In light of the established role of HAND2 in development and its high expression in SVF, we investigated adipose *Hand2* gene expression in the postnatal phase, 1 day, 2 weeks and 10 weeks after birth. *Hand2* levels were the highest in scWAT and BAT of 1-day-old pups and after 2 weeks in the gWAT, since this depot develops later than scWAT and BAT (ESM Fig. 2a,f,k). We also analysed the expression of *Nr3c1, Pref1* (also known as *Dlk1*), *Plin1* and *Adipoq* as markers of different stages of adipocyte differentiation (ESM Fig. 2b–e, g–j, l–o). Interestingly, chronic rosiglitazone treatment, which induces thermogenic adipocyte differentiation [3], did not affect *Hand2* expression in mouse cells (Fig. 2d). In adipocytes differentiated from hSVF, *HAND2* was higher in the classical white compared with the rosiglitazone-induced thermogenic differentiation regimen. *UCP1* expression was higher in thermogenic adipocytes, as expected (Fig. 2e,f). The hMADS cell model has been described as a reliable tool for studying the metabolism of white and thermogenic adipocytes [19, 20]. In hMADS cells, *HAND2* was also higher in the white compared with the thermogenic differentiation regimen (Fig. 2g,h). Furthermore, we confirmed that *HAND2* mRNA was induced early in hMADS adipogenesis (Fig. 2i). Interestingly, fully differentiated hMADS adipocytes showed a slightly higher expression of *HAND2* than pre-adipocytes (Fig. 2k). We confirmed the early induction of *Hand2* in mSVF (Fig. 2m) but could not find major change in *Hand2* gene expression between undifferentiated and differentiated cells (Fig. 2o). *PLIN1* was used as marker for mature adipocytes and showed higher level in differentiated adipocytes (Fig. 2j,l,n,p). On the contrary, *PREF1,* a marker of the pre-adipocyte stage was not expressed in hMADS but showed higher levels in undifferentiated mSVF (ESM Fig. 2p,q). In summary, these data illustrate that *HAND2* is highly and selectively expressed in white adipocytes with a spike of expression during the commitment phase towards the adipocyte lineage.

### Loss of HAND2 impairs adipogenesis

**Fig. 3:**
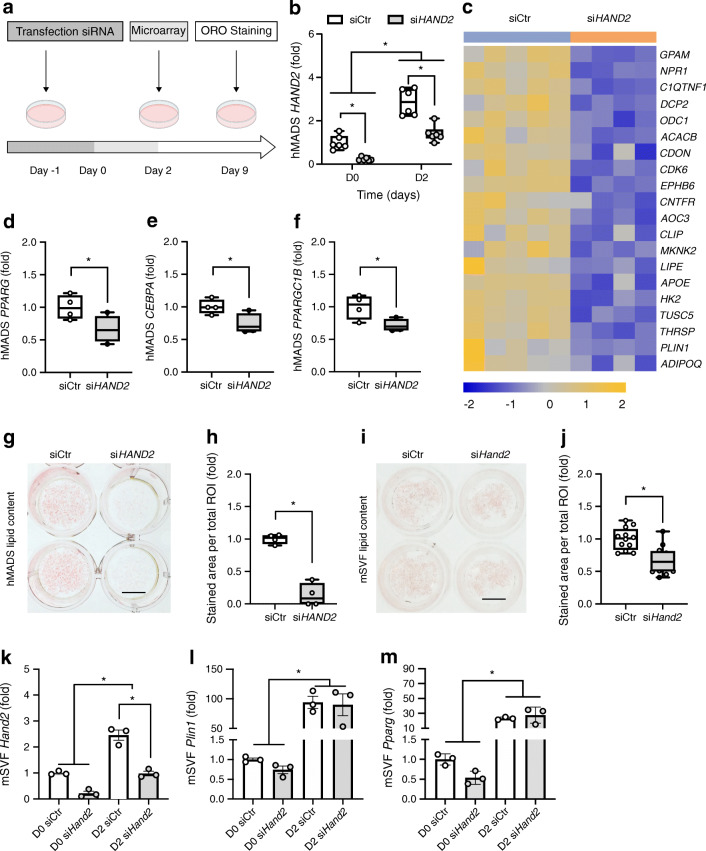
Loss of *HAND2* impairs adipocyte differentiation. (**a**) Experimental protocol. (**b**) *HAND2* mRNA expression was measured day 0 and day 2 (*n* = 6 replicates). (**c**) Heat map, generated from the microarray data, of the most inhibited genes (*n* = 4–5 replicates). (**d**–**f**) Gene expression of adipogenesis markers (*n* = 4 replicates). (**g**–**j**) Oil Red O staining of hMADS cells (**g**, **h**) (*n* = 4 replicates) and mSVF (**i**, **j**) transfected with si*Hand2* 2 days prior to induction and collected at day 6 (*n* = 12 replicates). (**k**–**m**) *Hand2* (**k**), *Plin1* (**l**) and *Pparg* (**m**) expression in mSVF transfected with si*Hand2* 2 days prior to induction and collected at day 0 and day 2 following the induction. Data were presented as fold change compared with the condition D0/siCtr (**b**, **k**–**m**); to the condition siCtr (**d**–**f**, **h**). Statistics: two-way ANOVA with Tukey test (**b**, **k**–**m**), two-tailed unpaired *t* test (**d**–**f**, **h**, **j**); median with upper and lower quartile ± maximum and minimum (**b**, **d**, **e**, **f**, **h**, **j**); mean ± SEM (**k**–**m**). Statistical significance is indicated by *p < 0.05. Ctr, control; D, day; ORO, Oil Red O; ROI, region of interest

As *HAND2* was induced in early adipogenesis in human and mouse adipocyte models, we hypothesised that HAND2 might be an important component of the adipogenesis program. To test this hypothesis, we silenced *HAND2* gene expression in hMADS cells before the induction of differentiation (Fig. 3a). *HAND2*-specific siRNA treatment led to diminished levels of *HAND2* mRNA at day 0 and day 2 compared with control siRNA-treated cells (Fig. 3b). Transcriptomic analysis at day 2 revealed that silencing of *HAND2* was associated with a marked downregulation of the expression of prominent mature adipocyte genes, including *ADIPOQ*, *APOE*, *LIPE* and *PLIN1* (Fig. 3c). Furthermore, key adipogenic transcription factors such as *PPARG*, *CEBPA* and *PPARGC1B* were expressed at lower levels (Fig. 3d–f), indicating that HAND2 was required for proper execution of adipogenesis. Ingenuity pathway analysis confirmed that silencing of *HAND2* led to a broad dysregulation of the transcriptional programs required for adipocyte biology (ESM Fig. 3a,b). Interestingly, among the in silico predicted inhibited upstream regulators were *NR3C1* (encoding the GR), *KLF15* and *PPARG*, all well-known master regulators of adipogenesis. In contrast, upstream regulators associated with proliferation such as *FGF2*, *TGFB* (also known as *TGFB1*) or *MIF* were predicted to be activated (ESM Fig. 3c). The aberrant execution of the transcriptional adipogenesis program was also mirrored in the overall cellular phenotype, as early *HAND2* silencing completely abolished the differentiation of hMADS cells into mature adipocytes as demonstrated by the lack of lipid droplet formation (Fig. 3g,h). Moreover, in mSVF-derived pre-adipocytes, early silencing of *Hand2* led to lower lipid droplet formation while the induction of key adipogenic genes, including *Plin1* and *Pparg*, remained largely intact (Fig. 3i–m). Taken together, our results indicate that HAND2 was required for adequate differentiation in human adipose-derived mesenchymal stem cells and modulated differentiation in mouse pre-adipocytes.

### *Hand2* in mature adipocytes is dispensable in vivo

**Fig. 4:**
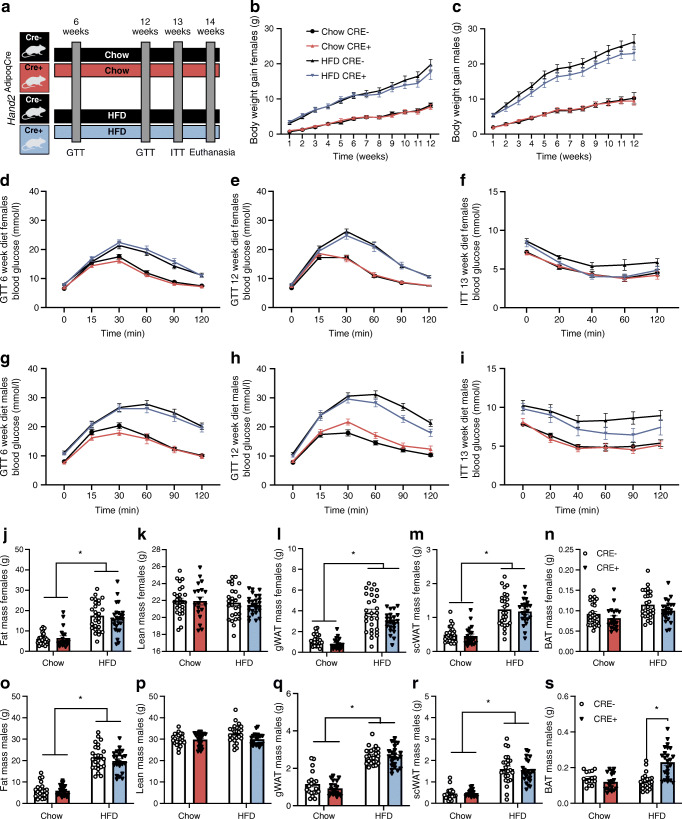
Metabolic phenotyping of *Hand2*^AdipoqCre^ mice fed an HFD. (**a**) *Hand2*^AdipoqCre^ (CRE+) and wild-type (CRE−) littermates, females and males, were fed an HFD (60%) for 6 or 12 weeks. Several metabolic variables were measured. (**b**, **c**) Body weight gain in females (**b**) and males (**c**). (**d**–**i**) GTT in females (**d**) and males (**g**) after 6 weeks of HFD diet; GTT and ITT in females (**e**, **f**) and males (**h**, **i**) after 12 weeks of HFD diet. (**j**–**s**) Final measurement of body composition (**j**, **k**, **o**, **p**) and adipose depot weight in females (**l**–**n**) and males (**q**–**s**). Each variable has been measured in three cohorts in females (Chow CRE− *n*=27 mice, Chow CRE+ *n*=20 mice, HFD CRE− *n*=27 mice, HFD CRE+ *n*=24 mice) and males (Chow CRE− *n*=21 mice, Chow CRE+ *n*=26 mice, HFD CRE− *n*=25 mice, HFD CRE+ *n*=23 mice). Statistics: two-way ANOVA with Tukey test; mean ± SEM (**b**–**s**). Statistical significance is indicated by *p < 0.05

In order to assess whether HAND2 might also be required for adipocyte function and systemic metabolic control in vivo, we created a conditional Cre-loxP mouse model for adipocyte-specific deletion of *Hand2*. We used an established transgenic mouse model, in which critical parts of the *Hand2* gene are flanked by loxP sites [24] and crossed this model with mice carrying Cre driven by the *Adipoq* promoter [26], which led to genetic deletion of *Hand2* in mature adipocytes in vivo (*Hand2*^AdipoqCre^) (ESM Fig. 4a,b). As adipocyte function is an important pillar of lipid homeostasis during fasting and the postprandial phase, we first tested plasma triacylglycerol and non-esterified fatty acid levels in chow-fed male and female mice. Fasted and refed plasma lipid levels remained largely unchanged between *Hand2*^AdipoqCre^ and wild-type littermate controls (ESM Fig. 4c–f). These observations were supported with human metabolic data showing no correlation between *HAND2* expression in WAT and plasma triacylglycerol levels (ESM Table 2). In addition, adipocytes are critically important for sustaining metabolic control in states of caloric excess, so we next tested the role of adipocyte HAND2 in DIO mice. Both male and female mice were placed on HFD for 12 weeks to induce weight gain and insulin resistance whereas chow diet-fed mice were included as controls (Fig. 4a). As expected, HFD feeding induced markedly higher weight gain compared with chow diet in both sexes, whereas the absence of *Hand2* in white adipocytes did not have an effect (Fig. 4b,c). During the study, we performed GTTs after 6 and 12 weeks as well as an ITT after 12 weeks of the feeding regimen. As expected, HFD-fed mice displayed markedly lower glucose tolerance as well as markedly higher insulin resistance relative to chow-fed controls at both time points, however, we did not detect any genotype-specific differences (Fig. 4d–i). At the end of the study, while lean mass remained unchanged across diets and genotypes, HFD-fed mice displayed markedly higher adiposity and WAT depot weights than chow-fed controls and these differences were independent of sex and genotype (Fig. 4j–s). A careful analysis of adipose tissue histology revealed the expected increase in adipocyte diameter in WAT upon HFD feeding, but overall WAT and BAT were phenotypically similar when comparing genotypes (ESM Fig. 5a–f). Following up on these in vivo findings, we also confirmed that *HAND2* is dispensable in differentiated hMADS cells in vitro as silencing of *HAND2* has no effect on lipid release (ESM Fig. 6a), glucose uptake (ESM Fig. 6b) or expression of key players of metabolic pathways (ESM Fig. 6c–m) or on phosphorylation of Akt under insulin stimulation (ESM Fig. 6n). In summary, while *HAND2* was required for proper adipogenesis in vitro, its silencing in vitro and in vivo in fully differentiated adipocytes did not affect the response to insulin stimulation or adipocyte function on glucose transport and lipolysis.

### HAND2 is regulated by GCs via the GR

**Fig. 5:**
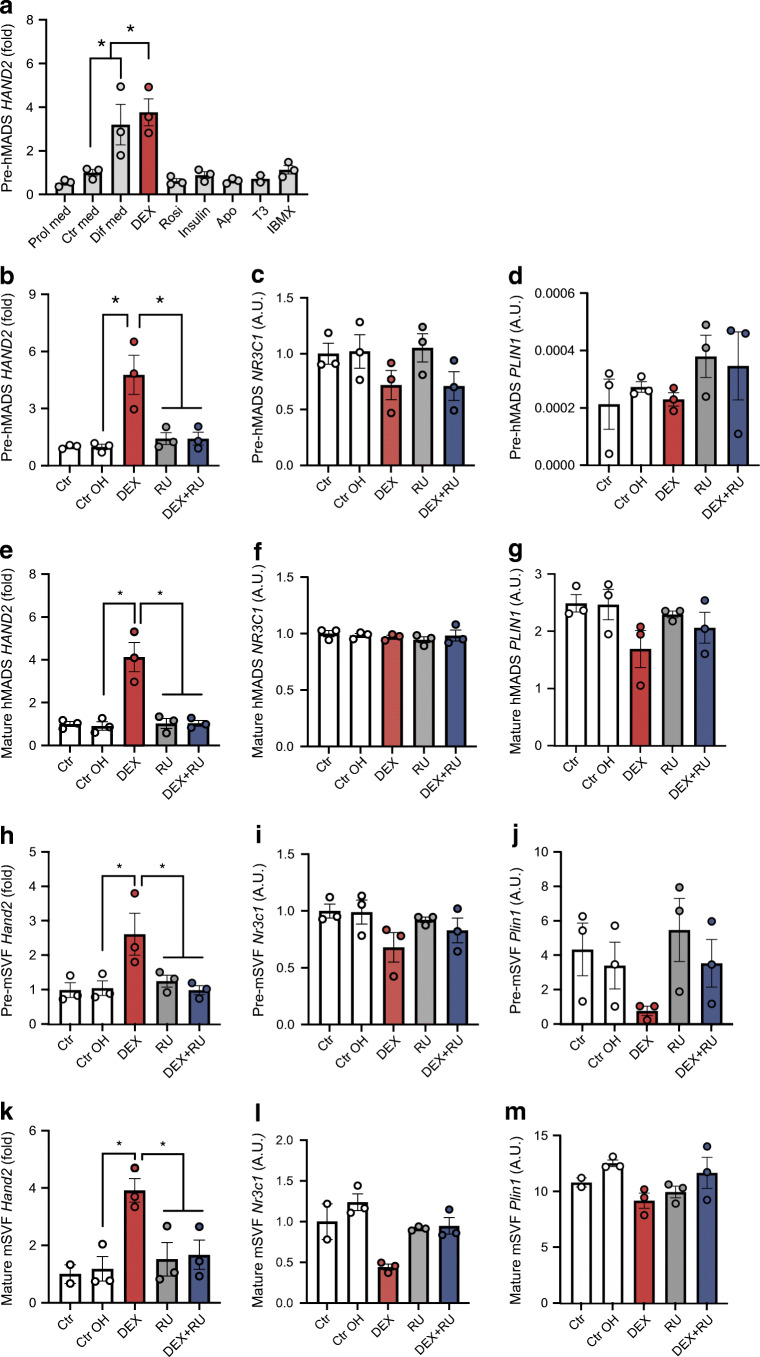
DEX regulates *HAND2* expression. (**a**) *HAND2* expression in hMADS pre-adipocytes. Proliferation media (DMEM 10% serum), control media (DMEM + F12), differentiation media (DMEM + F12 + 1 μmol/l DEX + 100 nmol/l rosiglitazone + 10 nmol/l insulin + 10 μg/ml apotransferrin + 0.2 nmol/l T_3_ + 500 μmol/l IBMX), rosiglitazone (DMEM + F12 + 100 nmol/l rosiglitazone), DEX (DMEM + F12 + 1 μmol/l DEX), insulin (DMEM + F12 + 10 nmol/l insulin), apo-transferrin (DMEM + F12 + 10 μg/ml apotransferrin), T_3_ (DMEM + F12 + 0.2 nmol/l T_3_), IBMX (DMEM + F12 + 500 μmol/l IBMX) (*n* = 3 replicates). (**b**–**g**) *HAND2, NR3C1* and *PLIN1* expression in hMADS pre-adipocytes (*n* = 3 replicates) (**b**–**d**) and mature adipocytes (*n* = 3 replicates) (**e**–**g**), treated with 2% ethanol, with DEX (1 μmol/l) and/or RU-486 (2 μmol/l) for 12 h (*n* = 3 replicates). (**h**–**m**) *Hand2, Nr3c1* and *Plin1* expression in mSVF pre-adipocytes (*n* = 3 replicates) (**h**–**j**) and mature adipocytes (*n* = 3 replicates) (**k**–**m**), treated with DEX (1 μmol/l) and/or RU-486 (2 μmol/l) for 12 h. Data are presented as fold change compared with the condition Ctr med (**a**), to the condition Ctr (**b**, **e**, **h**, **k**). Data are presented as arbitrary units representing copy number normalised to *TBP* (**c**, **d**, **f**, **g**, **i**, **j**, **l**, **m**). Statistics: one-way ANOVA with Tukey test; mean ± SEM. Statistical significance is indicated by *p < 0.05. Apo, apo-transferrin; Ctr, control; Ctr OH, 2% ethanol; Dif, differentiation; IBMX, isobutylmethylxanthine; med, media; Rosi, rosiglitazone; RU, RU-486

**Fig. 6:**
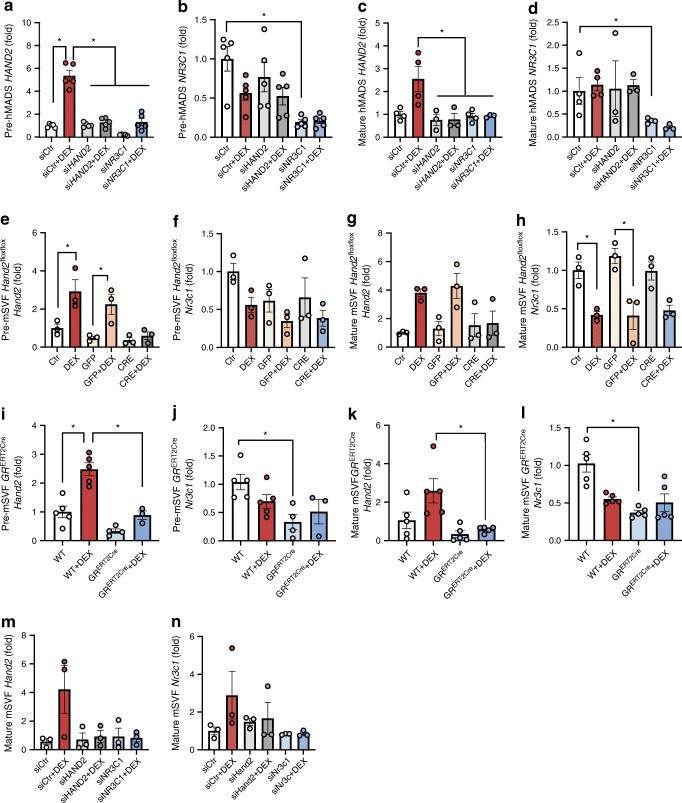
*HAND2* is regulated by GCs via the GR. (**a**–**d**) *HAND2* and *NR3C1* expression in hMADS pre-adipocytes (*n* = 5 replicates) (**a**,**b**) and mature adipocytes (*n* = 4 replicates) (**c**,**d**), transfected with si*HAND2* or si*NR3C1*, treated or not with DEX (1 μmol/l) for 12 h. (**e**–**h**) *Hand2* and *Nr3c1* expression in mSVF pre-adipocytes (*n* = 3 replicates) (**e**,**f**) and mature adipocytes (*n* = 3 replicates) (**g**,**h**) from *Hand2*^flox/flox^ mice. (**i**–**l**) *Hand2* and *Nr3c1* expression in mSVF in pre-adipocytes (*n* = 3–5 replicates) (**i**, **j**) and differentiated adipocytes (*n* = 5 replicates) (**k**,**l**), all treated with tamoxifen and treated or not with DEX (1 μmol/l) for 12 h from *GR*^ERT2Cre^ mice. (**m**,**n**) *Hand2* and *Nr3c1* expression in mSVF differentiated adipocytes and transfected with si*Hand2* or si*Nr3c1* 24 h before DEX treatment (1 μmol/l) for 12 h. Data are presented as fold change compared to the condition siCtr (**a**–**d**, **m,n**); to the condition Ctr (**e**–**h**), to the condition WT (**i**–**l**). Statistics: two-way ANOVA with Tukey test; mean ± SEM (**a**–**n**). Statistical significance is indicated by *p < 0.05, WT, wild-type

Our results supported the hypothesis that *HAND2* expression levels in adipocytes were determined early during adipogenesis, and that *HAND2* expression was required for differentiation of stem cells into adipocytes in vitro. Among the most powerful and established inducers of adipogenesis are insulin, cAMP-raising agents (e.g. isobutylmethylxanthine [IBMX]), agonists of PPARγ (e.g. rosiglitazone), and GR agonists, such as DEX. As HAND2 expression was induced in cell culture upon treatment with this commonly used adipogenic hormone cocktail, we explored the ability of the individual ingredients to regulate *HAND2*. We found that DEX, but not the other adipogenic agents, induced *HAND2* expression (Fig. 5a). This induction of *HAND2* by DEX was antagonised by RU-486 treatment in hMADS (Fig. 5b–g) as well as in SVF cells (Fig. 5h–m) irrespectively of whether it was added before or after differentiation. A time course of DEX and RU-486 treatment revealed that HAND2 expression peaked after 3 h of treatment and remained significantly different from control cells up to at least 24 h (ESM Fig. 7a) *NR3C1* and *PLIN1* were not affected by *HAND2* knockdown (ESM Fig. 7b,c). Of note, as expected, *PLIN1* expression was very low in pre-adipocytes, and markedly higher in differentiated adipocytes, and was not significantly affected by pharmacological manipulation of GR, with the only exception being mature mSVF *Hand2*^flox/flox^ (*p* < 0.05). *Pref1* showed higher levels in undifferentiated mSVF (ESM Fig. 7d,e). Furthermore, in mice, a single DEX injection led to higher *Hand2* expression in gWAT in females (ESM Fig. 8a,b). However, chronic DEX treatment induced an increase of energy expenditure in the light phase and a decrease in the dark phase; nevertheless no specific phenotype was observed in *Hand2*^AdipoqCre^ mice (ESM Fig. 8c,d,g). Other metabolic variables including activity, food intake, body weight, body composition and blood glucose concentration were not influenced by the absence of *Hand2* in mature adipocytes, neither at 22°C nor at 30°C thermoneutrality (ESM Fig. 8e,f,h–m). Interestingly, chronic DEX treatment by daily injections for 2 days or 2 weeks lowered *Hand2* levels in gWAT (ESM Fig. 8n,o). Similar findings were observed in humans receiving daily DEX treatment for more than a week for chronic inflammatory diseases. (ESM Fig. 8p). Congruent with a GR-dependent mechanism, siRNA-mediated knockdown of *NR3C1* in hMADS pre-adipocytes as well as mature adipocytes (Fig. 6a–d) abolished the effect of DEX on *HAND2.* Genetic inactivation of *Hand2* in SVF from gWAT of *Hand2*^flox/flox^ mice with Cre-encoding mRNA both in pre-adipocytes and mature adipocytes prevented *Hand2* increase in pre-adipocytes (condition GFP + DEX vs CRE + DEX *p*= 0.059) and mature adipocytes (condition GFP + DEX vs CRE + DEX *p* = 0.11) under DEX treatment but did not affect GR expression (Fig. 6e–h). In pre-adipocytes and mature adipocytes isolated from WAT SVF of *Nr3c1*^flox/flox^ mice crossed with mice expressing the global tamoxifen-inducible Cre-ERT2 fusion protein (*GR*^ERT2CRE^) [25], we confirmed that GR regulated *Hand2* expression as tamoxifen treatment abrogated the increase in *Hand2* under DEX stimulation (Fig. 6i–l). Using an RNA interference approach in mSVF we made similar observations (condition siCtr + DEX vs si*Hand2* + DEX *p*  =  0.09) (Fig. 6m–n). Furthermore, in all the conditions described above, as expected, *PLIN1* expression was almost nonexistent in pre-adipocytes compared with adipocytes and remained unaffected by the different treatments while *Pref1* expression was higher in undifferentiated cells (ESM Fig. 7f–p). Altogether these data support the notion that HAND2 is regulated by the GC–GR pathway during adipocyte differentiation.

### *Hand2* expression is necessary but insufficient for adipocyte differentiation

We explored whether increasing *Hand2* expression was sufficient to compensate for GCs at the beginning of differentiation. To this end, we overexpressed *Hand2* using a pcDNA-3XFlag-*Hand2* vector 24 h before inducing the differentiation of 3T3-L1 cells either using a classical cocktail of induction including DEX (1 μmol/l) or with a cocktail depleted of DEX. Comparing cells transfected with the control or *Hand2* vectors, we did not detect any significant difference in key differentiation markers, neither 24 h nor 6 days after induction (ESM Fig. 9a–h, k–q) and lipid accumulation was unchanged (ESM Fig. 9i–j). In conclusion, while HAND2 is required early in the transition of mesenchymal cells into the adipocyte lineage, its induction is insufficient for differentiation and unable to compensate for GC stimulation.

### Gene networks regulated by the GR–HAND2 pathway

**Fig. 7:**
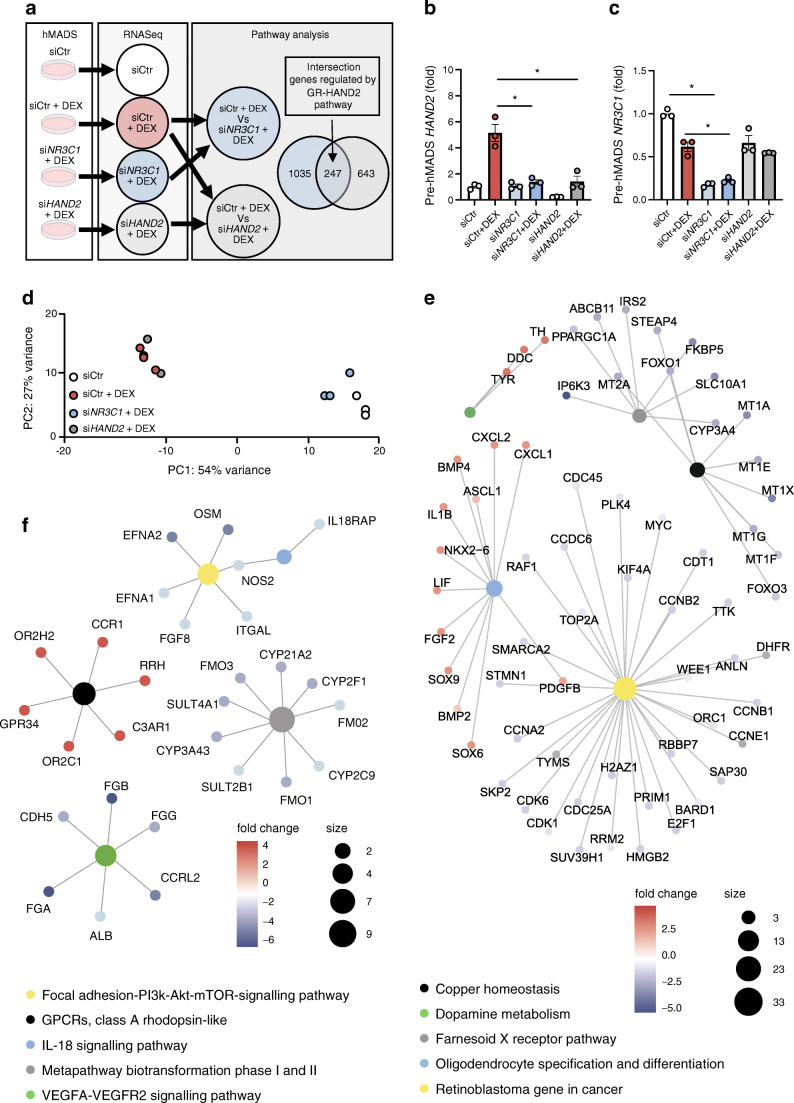
Gene networks during adipocyte differentiation regulated by the GR–HAND2 pathway. (**a**) Experimental protocol: hMADS pre-adipocytes were transfected with si*NR3C1* or si*HAND2* 36 h prior to a 12 h treatment with DEX (1 μmol/l). Pathway analysis was performed on the genes regulated by *NR3C1* and commonly regulated by *NR3C1* and *HAND2* (*n* = 3 replicates). (**b**–**c**) qPCR analysis of *HAND2* and *NR3C1* gene expression. (**d**) Principal component analyses of the RNAseq data. (**e**,**f**) GO pathway analysis of siCtr + DEX vs si*NR3C1* + DEX (**e**) and of the intersection between siCtr + DEX vs si*NR3C1* + DEX and siCtr + DEX vs si*HAND2* + DEX (**f**). Data are presented as fold change compared with the condition siCtr (**b**,**c**). Statistics: two-way ANOVA with Tukey test; mean ± SEM (**b**,**c**). Statistical significance is *p < 0.05. GPCR, G protein-coupled receptors; mTOR, mammalian target of rapamycin; PC, principal component; PI3K, phosphatidylinositol 3-kinase; VEGF, vascular endothelial growth factor

Our results indicate that HAND2 was induced by GR and required for adipocyte differentiation but dispensable in mature adipocytes. Therefore, we set out to understand the transcriptional networks during early adipogenesis downstream of the GR–HAND2 pathway. We silenced HAND2 and NR3C1 in hMADS cells and treated the cells 2 days later with DEX for 12 h and performed RNAseq (Fig. 7a–c). While the transcriptome signature of cells with NR3C1 silencing treated with DEX clustered together with the control samples without DEX treatment (Fig. 7d), cells with HAND2 silencing clustered with control treated with DEX, suggesting that HAND2 regulates only a small subset of GR-related genes. Using a gene set enrichment analysis [27, 28], we determined the transcriptional networks regulated by GC–GR signalling for benchmarking our dataset. Indeed, we found many confirmed GC–GR signalling pathways, underlining the reliability of our dataset (Fig. 7e). Focusing on gene expression levels that were differentially and commonly regulated by GR and HAND2 as part of the GR–HAND2 pathway we found four main pathways to be regulated: the metapathway biotransformation phase I and II, including genes from the cytochrome p450 family implicated in the synthesis of cholesterol steroids and other lipids, the mammalian target of rapamycin (mTOR) pathway and the large family of class A rhodopsin-like G protein-coupled receptors (GPCRs) and the signalling pathway vascular endothelial growth factor (VEGF)A–VEGFR2 (Fig. 7f). We confirmed the regulation of several of these gene sets in gWAT of *Hand2*^AdipoqCre^ mice including *Efna1*, *Efna2*, *Fmo2* and *Cyp2f1* (ESM Fig. 10a). Using WAT SVF from *Hand2*^3XFlag^ mice or the associated vector pcDNA-3XFlag-*Hand2* to overexpress HAND2 in 3T3-L1 cells, we investigated by ChIP qPCR whether HAND2 was directly binding putative targets that we selected from our RNASeq data (*Efna2*, *Clnd1*, *Rgs3* and *Rheb*), known targets of GR (*Per1* and *Gilz* [also known as *Tsc22d3*] or *Tbx2* and *Hand2* itself, which were already published to be targets of HAND2 [15]. We did not find evidence that HAND2 was enriched in the selected binding sites (ESM Fig. 10b–g). In summary, these data confirm that HAND2 plays an important role very early in the adipocyte differentiation process and might play an important role in the execution of the GC–GR program.

## Discussion

In this study, we show that HAND2 is a critical factor for early adipogenesis, regulated by GC–GR signalling and correlated to body weight and obesity in mice and humans. In hMADS cells, the earlier we manipulated *HAND2* expression, the more severe was the effect in interfering with proper adipogenesis, while in mSVF the effects were similar but much smaller in magnitude. These differences could be explained by the different level of commitment of hMADS compared with primary pre-adipocytes. Furthermore, while GCs are required for human adipocyte differentiation they are largely dispensable in mouse models [11]. Therefore, it should not come as a surprise that our conditional *Hand2* mouse model using *Adipoq*-Cre did not show any overt metabolic phenotype, as the *Adipoq* gene and hence the Cre recombinase are expressed relatively late in adipocyte differentiation [26]. One way to probe this further would be to deplete *Hand2* under a Cre driver specifically expressed in the commitment phase of the adipocytes, such as Wnt or hedgehog [29, 30]. Nevertheless, those master regulators are not specific to adipocytes and might lead to lethality in the very early stage of the embryo development similarly to the global deletion of *Hand2* [16, 17]. As *Hand1* gene expression was undetectable in adipocytes, it is unlikely that there is compensation of HAND2 loss by HAND1 activity in our studies.

The second major finding of our study is that HAND2 expression is regulated by GCs via GR during early and late stages of adipogenesis. Available data in the literature did not identify HAND2 as a direct target of GR [11, 31, 32], a finding that we confirmed by performing GR ChIP qPCR. We also investigated the *HAND2* promoter area and did not find potential interactions between *PPARG*, *PRDM16*, *CEBPA* and *CEBPB*. Our RNAseq results demonstrated that GR is required for the global effects of DEX, which was expected. In contrast, cells with loss of *HAND2* still had a relatively intact transcription profile, indicating that HAND2 only regulates a small and defined set of genes upon DEX treatment. Further analyses are required to interrogate the relevance of these putative downstream effectors of HAND2 for adipogenesis. Considering the pleiotropic effects of DEX and GR signalling in the body, it is possible that some of the previous functions attributed to HAND2, for example in the heart, also involve GR activity as well as, vice versa, that established GR-mediated effects, for example in immune cells, could also involve HAND2.

Finally, lower levels of *Hand2* in gWAT and visWAT observed under chronic DEX treatment might be explained by the induction of a regulatory feedback loop or by the length of the exposure. Furthermore, *HAND2* expression correlates with BMI in visWAT but not scWAT. While more work is needed to understand this depot-specific regulation, it might relate to 11β-hydroxysteroid dehydrogenase (HSD)-1, a key enzyme in GC metabolism that contributes to the increased levels of GCs specifically in visWAT of obese individuals [33–35]. Under these conditions, reactivation of *HAND2* expression in obesity could help stimulate adipogenesis and healthy adipose tissue expansion, thus restoring insulin sensitivity and metabolic health. In summary, our study introduces HAND2 as a novel player in adipogenesis and highlights a new layer of GC–GR signalling, thus enhancing our understanding of adipocyte biology in obesity.

## Supplementary Information

ESM (PDF 46729 kb)

## Data Availability

Array data have been submitted to the GEO database at NCBI (GSE148699). Other data that support the findings of this study are available upon reasonable request.

## Abbreviations

BAT: Brown adipose tissue
DEX: Dexamethasone
DIO: Diet-induced obesity
GC: Glucocorticoid
GR: Glucocorticoid receptor
gWAT: Gonadal white adipose tissue
HAND2: Heart and neural crest derivatives-expressed 2
HFD: High-fat diet
hMADS: Human multipotent adipose-derived stem (cell)
hSVF: Human stromal vascular fraction
mSVF: Mouse stromal vascular fraction
PPARγ: Peroxisome proliferator-activated receptor γ
qPCR: Quantitative PCR
RNASeq: RNA sequencing
scWAT: Subcutaneous white adipose tissue
SVF: Stromal vascular fraction
visWAT: Visceral white adipose tissue
WAT: White adipose tissue
