# Supplementary material for: HAND2 is a novel obesity-linked adipogenic transcription factor regulated by glucocorticoid signalling
Source: Diabetologia. 2021 May 20;64(8):1850–65. doi: 10.1007/s00125-021-05470-y (PMC8245394; doi:10.1007/s00125-021-05470-y)
Supplement: Supplementary file 1 — (PDF 46729 kb) [file 125_2021_5470_MOESM1_ESM.pdf]

Electronic Supplementary Material: Supplementary Methods

Giroud et al., 2021: HAND2 is a novel obesity-linked adipogenic transcription factor regulated by glucocorticoid signaling

Cell culture and stromal vascular fraction (SVF) preparation:

Human multipotent adipose derived stem (hMADS) cells were kindly provided by Z. Amri and were free of mycoplasma contamination [1, 2]. Cells were plated at a density of 5000-7000 cells/cm2 in Dulbecco's Modified Eagle's Medium (DMEM), (Lonza, Switzerland # BE12-707F) supplemented with 10% FBS (Sigma, Germany), 10 mmol/l 4-(2-hydroxyethyl)-1-piperazineethanesulfonic acid (HEPES) (Gibco, Germany #15630056), 2.5 ng/ml human fibroblast growth factor-basic (hFGF2) (PeproTech, Germany #100-18B), 50 mg/ml penicillin, and 50 mg/ml streptomycin (Gibco, Germany #15140122). Once the cells reached confluency (day -2), hFGF2 was removed. 48 hours later (day 0), the differentiation was induced using DMEM/Ham's F12 (Lonza, Switzerland # BE12-615F) supplemented with 10 mg/ml apo-transferrin (Sigma, Germany # T1428), 10 nmol/l insulin (Sigma, Germany # I 9278), 100 nmol/l rosiglitazone (Cayman, Germany #71740), 0.2 nmol/l triiodothyronine (T_3) (Sigma, Germany #6893-02-3), 1 mmol/l Dexamethason (DEX) (Sigma, Germany # D4902), 1 mmol/l 3-isobutyl-1-methylxanthine (IBMX) (Sigma, Germany #28822-58-4). 2 days later, the medium was changed to DMEM/Ham's F12 supplemented with 10 mg/ml transferrin, 10 nmol/l insulin, 0.2 nmol/l triiodothyronine and 1 µmol/l rosiglitazone. The medium was changed every second day until day 9. At day 9, the medium was changed either to the same cocktail (DMEM/Ham's F12 supplemented with 10 mg/ml transferrin, 10 nmol/l insulin, 0.2 nmol/l triiodothyronine and 1 µmol/l rosiglitazone) in order to differentiate the cells in thermogenic adipocytes, or to the same cocktail without rosiglitazone to obtain white adipocytes.

Mouse SVF (mSVF) was obtained from subcutaneous white adipose tissue (scWAT), brown adipose tissue (BAT) and gonadal WAT (gWAT). Tissues were minced and vigorously shaken at 37°C in DMEM combined with collagenase (Sigma, Germany #11088793001) and BSA (Sigma, Germnay # A8806-5G) media (1/3 ratio) until complete digestion of the tissue (30-45 minutes). The activity of the collagenase was inhibited by adding serum to reach 10% v/v serum final. The mix of cells, digestion medium and serum was then filtered through a 100 µm nylon mesh and centrifuged

for 10 minutes at 800 g. The supernatant was discarded, and the pellet resuspended in DMEM 10% v/v Serum and filtered through a 70 µm nylon mesh and centrifuged for 10 min at 800 g. Once again, the pellet was resuspended in DMEM 10% v/v serum and filtered through a 48 µm nylon mesh. The cells were then diluted in DMEM supplemented with 10% v/v FBS, 10 mmol/l HEPES, 50 mg/ml penicillin, 50 mg/ml streptomycin and 50mg/ml gentamycin. At day 0, the differentiation was induced using DMEM 10% v/v serum supplemented with 200 nmol/l insulin, 1 µmol/l rosiglitazone, 0.2 nmol/l triiodothyronine, 1 mmol/l DEX and 1mmol/l IBMX. From day 2 to day 8, the medium was changed every other day with DMEM 10% v/v serum supplemented with 200 nmol/l insulin.

Human SVF (hSVF) was isolated from scWAT, collected from healthy patients (abdominoplasty) and differentiated by administering the same adipogenic cocktail as for hMADS cells [3]. The study was approved by the University Ulm ethical committee (vote no. 300/16) and all patients gave written informed consent.

Gene expression and functional analysis in vitro:

20 nmol/l siRNA (SMARTpool ON-TARGETplus siRNA Horizon Discovery, UK) or 1 µg/ml of mRNA CRE (StemMACS Cre Recombinase mRNA Miltenyi Biotec, Germany #130-101-113) were used to silence *HAND2* in hSVF, hMADS and mSVF. 20 nmol/l siRNA was used to silence *NR3C1* in hMADS and mSVF. Transfection was performed using Lipofectamine RNAiMAX Transfection Reagent (Thermo Fisher, Germany # 13778075) following the manufacturer protocols. Cells were collected 48 hours after transfection. To over-express *Hand2* we infected mSVF or 3T3L1 cells with the pcDNA-3XFlag-*Hand2* vector [4] and the pENTR-CMV-MCS-TKpA (Addgene USA) as control using TransIT-X2 Dynamic Delivery System (Mirus, US # MIR 6003) . Cells were collected 72 hours after infection. Chemical activation and inhibition of GR was performed by 12 h treatment with 1 µmol/l DEX (DEX) (Merk, Germany #D4902,) and/or 2 µmol/l RU486 (Merk, Germany #475838), respectively.

Protein expression of AKT (Cell Signaling, Germany #9272, 1/1000) , pAKT (Cell Signaling, Germany #4051, 1/1000) and β-ACTIN (Cell Signaling, Germany #4970, 1/1000) was quantified by western blot as previously described [5]. Briefly, membranes were blocked and primary as well as secondary antibodies applied in 1% ROTI-Block (Roth, Germany #A151.1). The membranes were always washed 3 times for 10 min in TBS-T.

Oil Red O staining (Sigma, Germany #O1391), glycerol accumulation (Sigma, Germany #F6428) and glucose uptake (Abcam, UK #ab136955) was measured. Transcriptional analysis was performed using SYBR-based qPCR (Thermo Fisher, Germany #A25741) (primers listed in ECM Table 1), microarray (Human Clariom S arrays, Thermo Fisher Scientific, Germany) and RNAseq (performed by Novogene, UK).

For microarray expression profiling, total RNA was isolated employing the RNAeasy kit (Qiagen) using the small and large RNA protocols for animal tissue and cultured cells, including on-column DNase digestion. RNA quality was measured with an Agilent 2100 Bioanalyzer and only high-quality RNA (RIN>7) was used for microarray analysis. Total RNA (150 ng) was amplified using the WT PLUS Reagent Kit (Thermo Fisher Scientific Inc., Waltham, USA) and the resulting cDNA was hybridized on Human Clariom S arrays (Thermo Fisher Scientific). Staining and scanning (Gene Chip Scanner 3000 7G) was done according to manufacturer`s instructions. Transcriptome Analysis Console (TAC; version 4.0.0.25; Thermo Fisher Scientific) was used for quality control and to obtain annotated normalized SST-RMA gene-levels. Statistical analyses were performed by utilizing the statistical programming environment R [6, 7]. Gene-wise testing for differential expression was done with the limma t-test including batch correction and Benjamini-Hochberg (BH) multiple testing correction (FDR < 10%). To reduce background, gene sets were filtered for TAC Data Above Background p-values<0.05. Heat maps were generated in R. Pathway analyses were generated through the use of Ingenuity Pathway Analysis software (IPA®, QIAGEN Redwood City, www.qiagen.com/ingenuity) using Fisher`s Exact Test p-values (Diseases & Functions), BH-corrected p-values (Canonical Pathways), or z-scores (Upstream Regulators). Array data have been submitted to the GEO database at NCBI (GSE148699).

For RNAseq analysis, library preparation and sequencing were performed by Novogene. An amount of 800 ng of total RNA was used and controlled quantitatively and qualitatively with Agilent Bioanalyzer 2100 system. Sequencing libraries were generated using NEBNext® UltraTM RNA Library Prep Kit for Illumina® (NEB, USA) following manufacturer’s recommendations. Prepared PCR products were purified and size-selected on AMPure XP beads (Beckman #A63881). The prepared libraries were sequenced (paired-end) on an Illumina platform. Read counts post processing, statistical analysis and differential gene expression analysis were performed using

DESeq2 [8] in R [7]. In order to observe the overall effect of experimental covariates a two-dimensional PCA plot was employed. GO gene enrichment analysis of differentially expressed genes was performed using ClusterProfiler [9]. ChIP-qPCR was performed as previously described [10]. . 20 million cells per biological replicate were crosslinked with formaldehyde solution (1% v/v in PBS) for 15 min. Chromatin was isolated using a lysis buffer (150 mmol/l NaCl; 5 mmol/l EDTA; 5 mmol/l Tris; 1% Triton; 0.5% NP40), and then sonicated in the shearing buffer (1% SDS; 10 µmol/l EDTA; 0.05 mmol/l Tris). Immunoprecipitations were performed by adding 3 µg of the following antibodies: αIgG (Cell Signaling Technology #2729); αGlucocorticoid Receptor (ProteinTech, Germany #24050-1-AP), αFLAG (Sigma, Germany #F1804) and sepharose protein A/G beads (Biomol, Germany #PAG50-00-0002) in a dilution buffer (0.1 g/L SDS, 1.10 g/L Triton, 1.2 µmol/l EDTA, 16.7 µmol/l Tris, 0.167 mol/l NaCl). DNA was eluted and qPCR was performed to validate the binding using the following primers (Supplementary table 1). All the experiments were performed at least 3 times.

Mouse experiments:

All animal studies were conducted in accordance with German animal welfare legislation and protocols were approved by the state ethics committee and government of Upper Bavaria (nos. ROB-55.2-2532.Vet_02-16-117, ROB-55.2-2532.Vet_02-17-125, ROB-55.2-2532.Vet_02-15-164). All mice were group-housed maintained in a climate-controlled environment at 22 °C with a 12-h dark–light cycle under specific pathogen-free conditions in the animal facility of the Helmholtz Center Munich. db/db Mice (JAX Mice Strain) were purchased from Charles River (https://www.criver.com/products-services/find-model/jax-dbdb-mice?region=23). Adipocyte-specific *Hand2* knockout mice (*Hand2*^{AdipoqCre}) were generated by crossing AdipoqCRE mice (Jackson laboratory, stock number 028020; C57BL/6J) with *Hand2*^{floxflox} mice (NMRI strain) kindly provided by R. Zeller [11]. *Hand2*^{AdipoqCre} (CRE+) and wild type littermates (CRE-) were used for all experiments. Animals were fed a high-fat diet (HFD) 60 kcal % fat (D12492, Research Diets Inc., New Brunswick, NJ, USA) ad libitum from the age of 6 weeks for 12 weeks after which glucose and insulin tolerance tests was performed. Animals were fasted at 8 am for 4 to 6 hours and subsequently injected intraperitoneally (i.p.) with glucose at 2 g/kg or insulin 0.8 U/kg

(HUMINSULIN Normal 100, Lilly Germany #PA257FSGE05). Blood samples were taken from the tail vein before and 15, 60, 90 and 120 min after the i.p. injection to assess glucose levels. (AccuChek Performa glucose meter, Roche Diabetes Care, Mannheim, Germany). Assessment of the fat mass vs lean mass was performed using whole-body magnetic resonance analysis (EchoMRI).

Histological analysis of the samples has been performed as following: The samples were fixed in 4% w/v neutrally buffered formalin and subsequently embedded in paraffin. 3 µm thick sections were stained with hematoxylin and eosin (HE), using a HistoCore SPECTRA ST automated slide stainer (Leica, Germany) with prefabricated staining reagents (Histocore Spectra H&E Stain System S1, Leica, Germany), according to the manufacturer's instructions. Stained tissue sections were scanned with an AxioScan. Z1 digital slide scanner (Zeiss, Jena, Germany) equipped with a 20x magnification objective. Quantification of lipid amount was morphometrically determined by automatic digital image analysis using the commercially available software Definiens Developer XD 2 (Definiens AG, Germany). The calculated parameter for BAT was the ratio of total area of lipid droplets per whole tissue section and for WAT the mean size of lipid droplets per whole tissue section. The experiment has been performed on 3 different cohorts of littermates with a total of 20 to 28 animals per group.

Wild type C57BL/6J (C57BL/6J (JAX Mice Strain), Charles River https://www.criver.com/products-services/find-model/jax-c57bl6j-mice) were intraperitoneally injected with 1 mg/kg DEX (Sigma, Germany # D9184-5G), for 6 hours, or daily for 2 days or 2 weeks.Indirect calorimetry, including energy expenditure, food consumption, oxygen consumption and locomotor activity, was measured using metabolic cages (TSE PhenoMaster cages TSE Systems, Bad Homburg, Germany) from *Hand2*^{AdipoqCre} (CRE+) and wildtype female littermates (CRE-) for three subsequent days where the animals were injected with DEX intraperitoneally (1 mg/kg) every morning at 8 am. The animals were acclimatized to metabolic cages for 1 week. The experiment was performed once with 6 animals per group. *GR*$^{flox/flox}$, *GR*^{ERT2CRE} and *Hand2*^{3XFlag} mice were generated as previously described [4, 12]. *Hand2* expression in wildtype mice versus diet-induced obese mice was measured in samples from 18 weeks old (10 animals per group) C57BL/6J males (C57BL/6J (JAX Mice Strain), Charles River https://www.criver.com/products-services/find-model/jax-c57bl6j-mice) fed chow or HFD. To correlate *Hand2* mRNA expression to body weight,

54 wild type C57BL/6J (C57BL/6J (JAX Mice Strain), Charles River https://www.criver.com/products-services/find-model/jax-c57bl6j-mice) and NMRI (*Hand2*^{AdipoqCre} (CRE-) male mice (18 to 24 weeks old) fed ad libitum with regular rodent chow were used. At termination of all experiments, the animals were killed by cervical dislocation and organs were collected and immediately frozen in liquid nitrogen and stored at -80 °C for further processing. The expression of *Hand2* (and other genes) was analyzed using SYBR-based qPCR (Thermo Fisher, Germany #A25741) (primers listed in ESM Table 1).

Human studies:

Human BAT was collected from FDG-PET-positive scan sites in the supraclavicular localization and subcutaneous WAT was taken from the same incision. The Study protocol was approved by the ethics committee of the Hospital District of Southwestern Finland, and participants provided written informed consent following the committee's instructions. The study was conducted according to the principles of the Declaration of HelsinkiparticipantsThe metabolic status of all potential participants who donated BAT was assessed, and only those with normal glucose tolerance and normal cardiovascular status (as assessed on the basis of electrocardiograms and measured blood pressure) were included. The age range of the participants was 23–49 years. We studied a group of 7 healthy volunteers (2 men and 5 women).

Human cohort 1 refers to human scWAT versus visWAT samples collected in the context of a cross-sectional study of 318 individuals (249 women, 69 men; BMI range: 21.9 – 97.3 kg/m², age range: 19-75 years). Additional 13 individuals who received DEX orally prior to the surgery for chronic inflammatory diseases, were compared to BMI matched (35 - 67 kg/m²) individuals from cohort 1. The dose varied between 1 mg and 4 mg DEX once daily for a period of one to two weeks for 11 patients and more than 14 days at a dose of 2 mg daily for the 2 additional patients. Abdominal omental (visWAT) and subcutaneous WAT samples were collected during elective laparoscopic abdominal surgery as described previously [13]. Adipose tissue was immediately frozen in liquid nitrogen and stored at -80 °C. The study was approved by the Ethics Committee of the University of Leipzig (approval no: 159-12-21052012) and performed in accordance to the declaration of Helsinki. All participants gave written informed consent before taking part in this study. Measurement of body composition and metabolic parameters was performed as described previously [13, 14].

Human cohort 2 refers to 96 individuals selected from the Leipzig Obesity Biobank to define age- and sex-matched groups of healthy lean individuals (MUO) (n=32; mean BMI: 23.4±1.5kg/m² mean age 43.6±7.1 years, 23 females, 9 males), individuals with metabolically healthy obesity (MHO) (n=32; mean BMI: 45.9±6.8kg/m² mean age 42.6±9.3 years, 23 females, 9 males) further BMI-matched to 32 individuals with obesity and type 2 diabetes (mean BMI: 45.3±4.7kg/m² mean age 42.7±6.7 years, 25 females, 7 males). Insulin sensitive and insulin resistant MHO as well as MUO subgroups were matched for BMI and body fat mass. Definition of the MHO subgroup has been described recently [15]. All individuals fulfilled the following inclusion criteria: 1) men or premenopausal women, 2) age >18years, 3) stable body weight, defined as the absence of fluctuations of >3% of body weight for ≥3 months before blood tests. In addition, the following exclusion criteria have been defined: 1) any acute or chronic inflammatory disease or symptoms of infection; 2) clinical evidence of either cardiovascular or peripheral artery disease; 3) smoking; 4) LDL-cholesterol > 4mmol/l; 5) any type of malignant disease; 6) thyroid dysfunction; 7) Cushing's disease or hypercortisolism; 8) alcohol or drug abuse; 9) pregnancy. The study was approved by Ethics committee of the University of Leipzig (approval number: 159-12-21052012) and all participants gave written informed consent before taking part in the study.

To analyze *HAND2* mRNA expression in human adipose tissue, RNA from visWAT and scWAT was extracted by using the RNeasy Lipid tissue Mini Kit (Qiagen, Hilden, Germany). Quantity and integrity of RNA was monitored with a NanoVue plus Spectrophotometer (GE Healthcare, Freiburg, Germany). 1 µg total RNA from subcutaneous and visceral adipose tissue were reverse-transcribed with standard reagents (Life technologies, Darmstadt, Germany). cDNA was then processed for TaqMan probe-based quantitative real-time polymerase chain reaction (qPCR) using the QuantStudio 6 Flex Real-Time PCR System (Life technologies, Darmstadt, Germany). The expression of HAND2 was calculated by standard curve method and normalized to the expression of hypoxanthine guanine phosphoribosyltransferase 1 (*HPRT1*) as the housekeeping gene. The probes (Life technologies, Darmstadt, Germany) for *HAND2* (Hs01047149_m1 and Hs00232769_m1) and *HPRT1* (Hs01003267_m1) span exon-exon boundaries to improve the specificity of the qPCR.

Statistics:

Data presented as bar chart were expressed as mean ± standard error of the mean (s.e.m.). Data presented as box and whiskers plot were shown as median with upper and lower quartile as well as maximum and minimum. Two-tailed, unpaired t-Test was used when comparing two conditions. 1-way ANOVA and 2-ANOVA with Tukey test were used when comparing three or more groups as reported in the figure legends. Analysis was performed using GraphPad Prism. $P < 0.05$ was considered significant as indicated by asterisks in the figure's legends. Mice experiments involving *Hand2*^{AdipoqCre} were performed 3 independent times and pooled. Mice were excluded for poor body condition or if dying before the end of the experiment. For the quantification of *HAND2* in control patients versus DEX treated patients, only patients in the same range of BMI were considered and outliers were excluded using the ROUT test Q1% from GraphPad Prism.

ESM Bibliography:

1. Elabd C, Chiellini C, Massoudi A, et al (2007) Human adipose tissue-derived multipotent stem cells differentiate in vitro and in vivo into osteocyte-like cells. Biochemical and Biophysical Research Communications 361(2):342–348. https://doi.org/10.1016/j.bbrc.2007.06.180

2. Rodriguez A-M, Elabd C, Delteil F, et al (2004) Adipocyte differentiation of multipotent cells established from human adipose tissue. Biochemical and Biophysical Research Communications 315(2):255–263. https://doi.org/10.1016/j.bbrc.2004.01.053

3. Pisani DF, Beranger GE, Corinus A, et al (2016) The K^+ channel TASK1 modulates β-adrenergic response in brown adipose tissue through the mineralocorticoid receptor pathway. FASEB j 30(2):909–922. https://doi.org/10.1096/fj.15-277475

4. Osterwalder M, Speziale D, Shoukry M, et al (2014) HAND2 targets define a network of transcriptional regulators that compartmentalize the early limb bud mesenchyme. Dev Cell 31(3):345–357. https://doi.org/10.1016/j.devcel.2014.09.018

5. Bartelt A, Widenmaier SB, Schlein C, et al (2018) Brown adipose tissue thermogenic adaptation requires Nrf1-mediated proteasomal activity. Nat Med 24(3):292–303. https://doi.org/10.1038/nm.4481

6. Rainer J, Sanchez-Cabo F, Stocker G, Sturn A, Trajanoski Z (2006) CARMAweb: comprehensive R- and bioconductor-based web service for microarray data analysis. Nucleic Acids Research 34(Web Server):W498–W503. https://doi.org/10.1093/nar/gkl038

7. R Development Core Team (2010) a language and environment for statistical computing: reference index. R Foundation for Statistical Computing, Vienna
8. Anders S, Huber W (2010) Differential expression analysis for sequence count data. Genome Biol 11(10):R106. https://doi.org/10.1186/gb-2010-11-10-r106
9. Yu G, Wang L-G, Han Y, He Q-Y (2012) clusterProfiler: an R Package for Comparing Biological Themes Among Gene Clusters. OMICS: A Journal of Integrative Biology 16(5):284–287. https://doi.org/10.1089/omi.2011.0118
10. Uhlenhaut NH, Barish GD, Yu RT, et al (2013) Insights into negative regulation by the glucocorticoid receptor from genome-wide profiling of inflammatory cistromes. Mol Cell 49(1):158–171. https://doi.org/10.1016/j.molcel.2012.10.013
11. Galli A, Robay D, Osterwalder M, et al (2010) Distinct Roles of Hand2 in Initiating Polarity and Posterior Shh Expression during the Onset of Mouse Limb Bud Development. PLoS Genet 6(4):e1000901. https://doi.org/10.1371/journal.pgen.1000901
12. Rapp AE, Hachemi Y, Kemmler J, Koenen M, Tuckermann J, Ignatius A (2018) Induced global deletion of glucocorticoid receptor impairs fracture healing. FASEB J 32(4):2235–2245. https://doi.org/10.1096/fj.201700459RR
13. Klöting N, Fasshauer M, Dietrich A, et al (2010) Insulin-sensitive obesity. American Journal of Physiology-Endocrinology and Metabolism 299(3):E506–E515. https://doi.org/10.1152/ajpendo.00586.2009
14. Langhardt J, Flehmig G, Klöting N, et al (2018) Effects of Weight Loss on Glutathione Peroxidase 3 Serum Concentrations and Adipose Tissue Expression in Human Obesity. Obes Facts 11(6):475–490. https://doi.org/10.1159/000494295
15. Mulhem A, Moulla Y, Klöting N, et al (2020) Circulating cell adhesion molecules in metabolically healthy obesity. Int J Obes (Lond). https://doi.org/10.1038/s41366-020-00667-4

Electronic Supplementary Material: Supplementary Tables

Giroud et al., 2021: HAND2 is a novel obesity-linked adipogenic transcription factor regulated by glucocorticoid signaling

ESM Table. 1:

Sequence of primers for SYBR-based qPCR.

Gene	Sequence
m_Hand2_F	TCCAAGATCAAGACACTGCG
m_Hand2_R	TCTTCTTGATCTCCGCCTTG
m_Nr3c1_F	AGCTCCCCCTGGTAGAGAC
m_Nr3c1_R	GGTGAAGACGCAGAAACCTTG
m_Plin1-F	CAAGCACCTCTGACAAGGTTC
m_Plin1-R	GTTGGCGGCATATTCTGCTG
m_Tbp_F	ACCCTTCACCAATGACTCCTATG
m_Tbp_R	ATGATGACTGCAGCAAATCGC
m_Hprt_F	TCAGTCAACGGGGGACACATAAA
m_Hprt_R	GGGGCTGTACTGCTTAACCAG
h_HAND2_F	GACACTCCCGTGTGGTAAGG
h_HAND2_R	AAGGGGTTGAGTAGGTTGGC
h_PLIN1_F	ACCCCCCTGAAAAGATTGCTT
h_PLIN1_R	GATGGGAACGCTGATGCTGTT
h_NR3C1_F	AAAGAGACGAATGAGAGTCCTTGGA
h_NR3C1_R	GCTTGCAGTCCTCATTCGAGTTT
h_TBP_F	ACGCCAGCTTCGGAGAGTTC
h_TBP_R	CAAACCGCTTGGGATTATATTCG
h_36B4_F	TGCATCAGTACCCCATTCTATCAT
h_36B4_R	AGGCAGATGGATCAGCCAAGA

Sequence of primers for CHIP qPCR.

DNA	Sequence
Cldn12_Chip_F	CCTCCGAGGAGTGTGGAG
Cldn12_Chip_R	GGTCAGCGTTACACAACCAG
Efna2_Chip_F	GCCAAGAGTGGGTACCTGTC
Efna2_Chip_R	GATATCTGGCCGAGTCCTCTC
Rgs3_Chip_F	CGGAAGTAGCCTAGTTAACAAAGTG
Rgs3_Chip_R	AAAAGAAGAGCGTCATGCTGTA
Rheb_Chip_F	GCCTTTTAAGACCCCACTGC
Rheb_Chip_R	GAGAGTCGTCTTGATGTCTGTGAC
Foxl2_Chip-F	GCTGGCAGAATAGCATCCG
Foxl2_Chip-R	TGATGAAGCACTCGTTGAGGC
Per1_Chip-2-F	TGGAACATCCTGTTCTCAGCG
Per1_Chip-2-R	AAGGAAGGCTGTGGCCAAC
Gilz_Chip-F	GGAGTTGGGTTCTGCCTGAG
Gilz_Chip-R	CCTGACCCTGTCTTTCCAGTG
Hand2_Chip_F	GCTTAGTCGCCTTCTCATTG
Hand2_Chip_R	TAGAGGCTCCCAGTTCTTTG
Tbx2_Chip_F	AGGGGAAGGAGGTTAGATGG
Tbx2_Chip_R	GCCTGGGAAGAAAGGCTTC

ESM Table. 2

a

Cohort 1	*HAND2*			
	visWAT		scWAT	
	r	P	*r*	P
Anthropometric data				
Body weight (kg)	-0,1963	**0,0004**	-0,1164	**0,0386**
BMI (kg/m²)	-0,2545	**<0.0001**	-0,083	0,1368
Metabolic data				
FPG (mmol/l)	-0,1137	**0,0433**	-0,004	0,9429
FPI (pmol/l)	-0,032	0,5682	-0,1732	**0,002**
HOMA-IR	-0,06214	0,2716	-0,1622	**0,0039**
Total cholesterol (mmol/l)	-0,03918	0,6683	0,2444	**0,0064**
HDL-C (mmol/l)	-0,03354	0,714	0,03375	0,7121
LDL-C (mmol/l)	-0,03896	0,674	0,1869	**0,041**
TG (mmol/l)	-0,09665	0,2876	0,01051	0,9078

b

Cohort 2	*HAND2*			
	visWAT		scWAT	
	r	P	*r*	P
Anthropometric data				
Body weight (kg)	-0,2489	**0,0145**	-0,36	**0,0003**
BMI (kg/m²)	-0,2108	**0,0393**	-0,3368	**0,0008**
Metabolic data				
FPG (mmol/l)	-0,1865	0,0704	-0,09636	0,3529
FPI (pmol/l)	-0,2534	**0,0166**	-0,1446	0,1765
HOMA-IR	-0,2306	**0,0238**	-0,1599	0,1196
Total cholesterol (mmol/l)	0,04394	0,6987	-0,01525	0,8932
HDL-C (mmol/l)	0,2664	**0,0111**	0,06913	0,5173
LDL-C (mmol/l)	0,09208	0,3881	-0,07369	0,4901
TG (mmol/l)	-0,02017	0,8572	0,04431	0,6927

ESM Table. 2 Correlation analysis between HAND2 expression in visWAT and scWAT and clinical data. (**a, b**) *HAND2* expression in human visWAT vs scWAT from cohort 1 (**a**) (n=318 participants) and cohort 2 (**b**) (n=96 participants) correlated with anthropometric and metabolic parameters. Statistics: Pearson correlation coefficient was computed. *r* (Pearson r), p (p value). FPG, fasting plasma glucose; FPI, fasting plasma insulin; HOMA-IR, homeostatic model assessment for insulin resistance; HDL-C, high-density lipoprotein cholesterol; low-density lipoprotein cholesterol; TG, triglycerides.

Electronic Supplementary Material: Supplementary Figures

Giroud et al., 2021: HAND2 is a novel obesity-linked adipogenic transcription factor regulated by glucocorticoid signaling

ESM Fig. 1

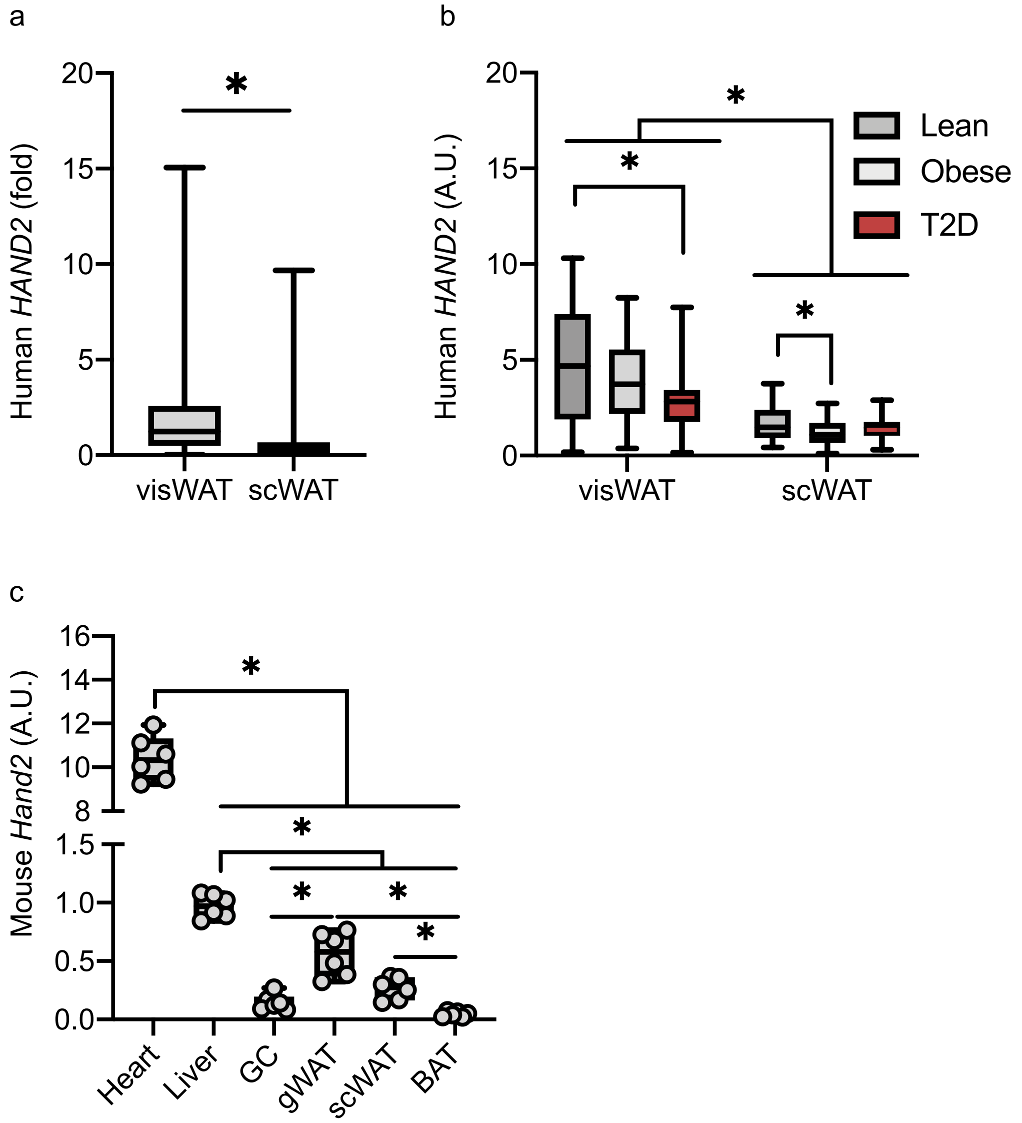

a
b
c
Human HAND2 (fold)
20
15
10
5
0
*
visWAT
scWAT
Human HAND2 (A.U.)
Lean
Obese
T2D
Mouse Hand2 (A.U.)
16
14
12
10
8
1.5
1.0
0.5
0.0
Heart
Liver
GC
gWAT
scWAT
BAT

ESM Fig. 1 *HAND2* expression in human adipose tissues and metabolic organs. **(a)** *HAND2* expression in visWAT and scWAT independently of the health status of the patients from cohort 1 (n=318 individuals). **(b)** *HAND2* expression in visWAT and scWAT grouped by BMI and insulin resistance status from cohort 2 (lean n=32 individuals, obese n=32 individuals, T2D n=32 individuals). **(c)** *Hand2* expression in different metabolic organs from mice (n=12 mice). Data are presented as fold change compared to the condition visWAT (**a**). Statistics: two-tailed unpaired t-test (**a**), two-way ANOVA with Tukey test (**b**), one-way ANOVA with Tukey test (**c**); median with upper and lower quartile ± maximum and minimum. Statistical significance is indicated by *$p<0.05$. T2D, type 2 diabetes.

ESM Fig. 2

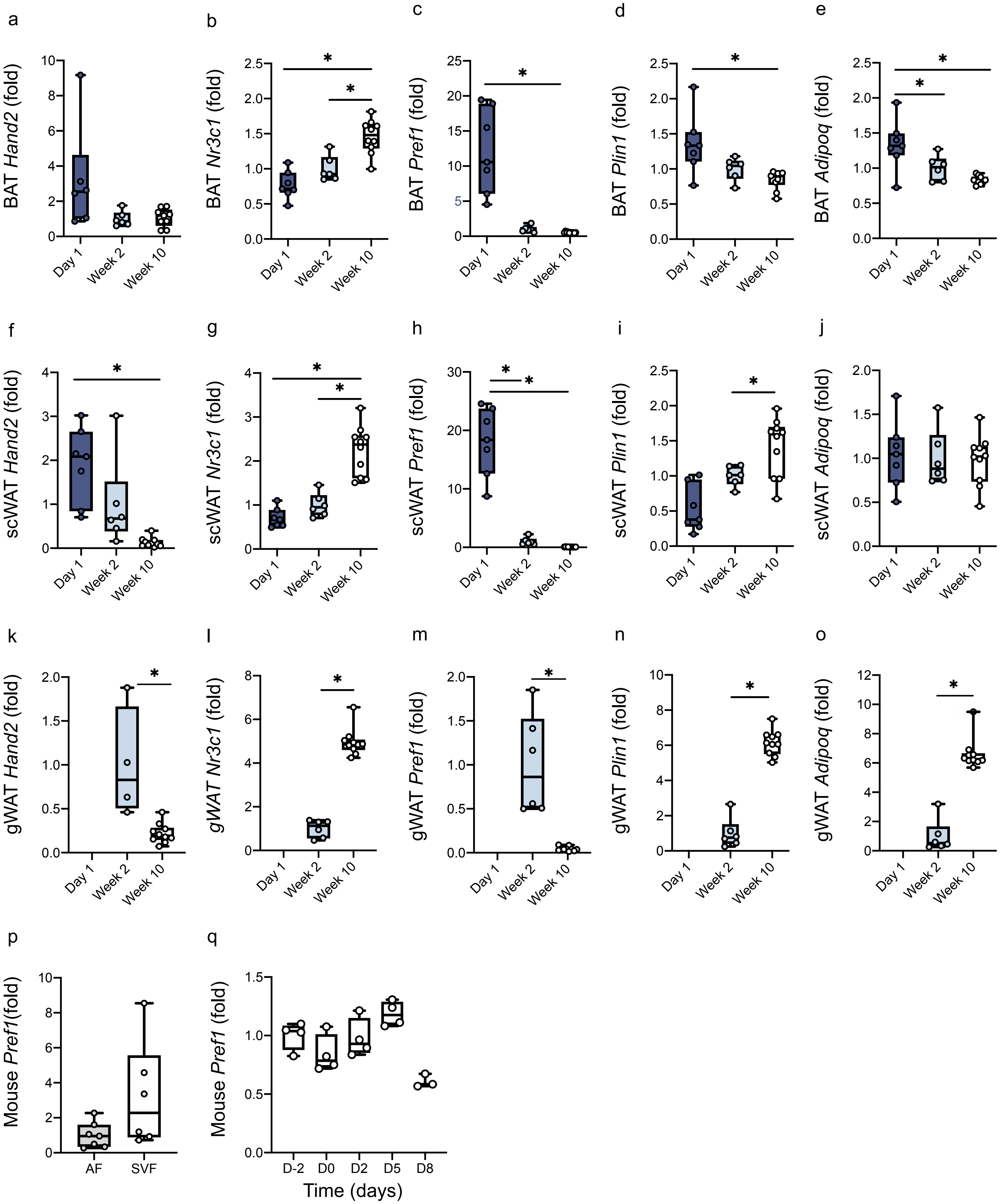
a
BAT Hand2 (fold)
b
BAT Nr3c1 (fold)
c
BAT Pref1 (fold)
d
BAT Plin1 (fold)
e
BAT Adipoq (fold)
f
scWAT Hand2 (fold)
g
scWAT Nr3c1 (fold)
h
scWAT Pref1 (fold)
i
scWAT Plin1 (fold)
j
scWAT Adipoq (fold)
k
gWAT Hand2 (fold)
l
gWAT Nr3c1 (fold)
m
gWAT Pref1 (fold)
n
gWAT Plin1 (fold)
o
gWAT Adipoq (fold)
Day 1
Week 2
Week 10
p
Mouse Pref1(fold)
AF
SVF
q
Mouse Pref1 (fold)
D-2
D0
D2
D5
D8
Time (days)

ESM Fig. 2 Characterization of HAND2 and other markers in cell differentiation and in adipose tissue during the post-natal phase. (**a, o**) Hand2, Nr3c1, Pref1, Plin1 and Adipoq expression in BAT (**a-e**), scWAT (**f-j**) and gWAT (**k-o**) 1 day, 2 weeks and 10 weeks after birth of *HAND2*$^{flox/flox}$ pups. Day 1 (n=6 mice), week 2 (n=6 mice), week 10 (n=10 mice). **(p, q)** *Pref1* expression in mouse adipose fraction versus stroma vascular fraction **(p)** and in a kinetic of differentiation of mSVF **(q)**. Data are presented as fold change compared to the condition week 2 (**a-q**), to the condition AF (**p**) and to the condition D-2 (**q**). Statistics: two-tailed unpaired t-test (**a, m-q**), one-way ANOVA with Tukey test (**b-l**); median with upper and lower quartile ± maximum and minimum. Statistical significance is indicated by *$p<0.05$.

ESM Fig. 3

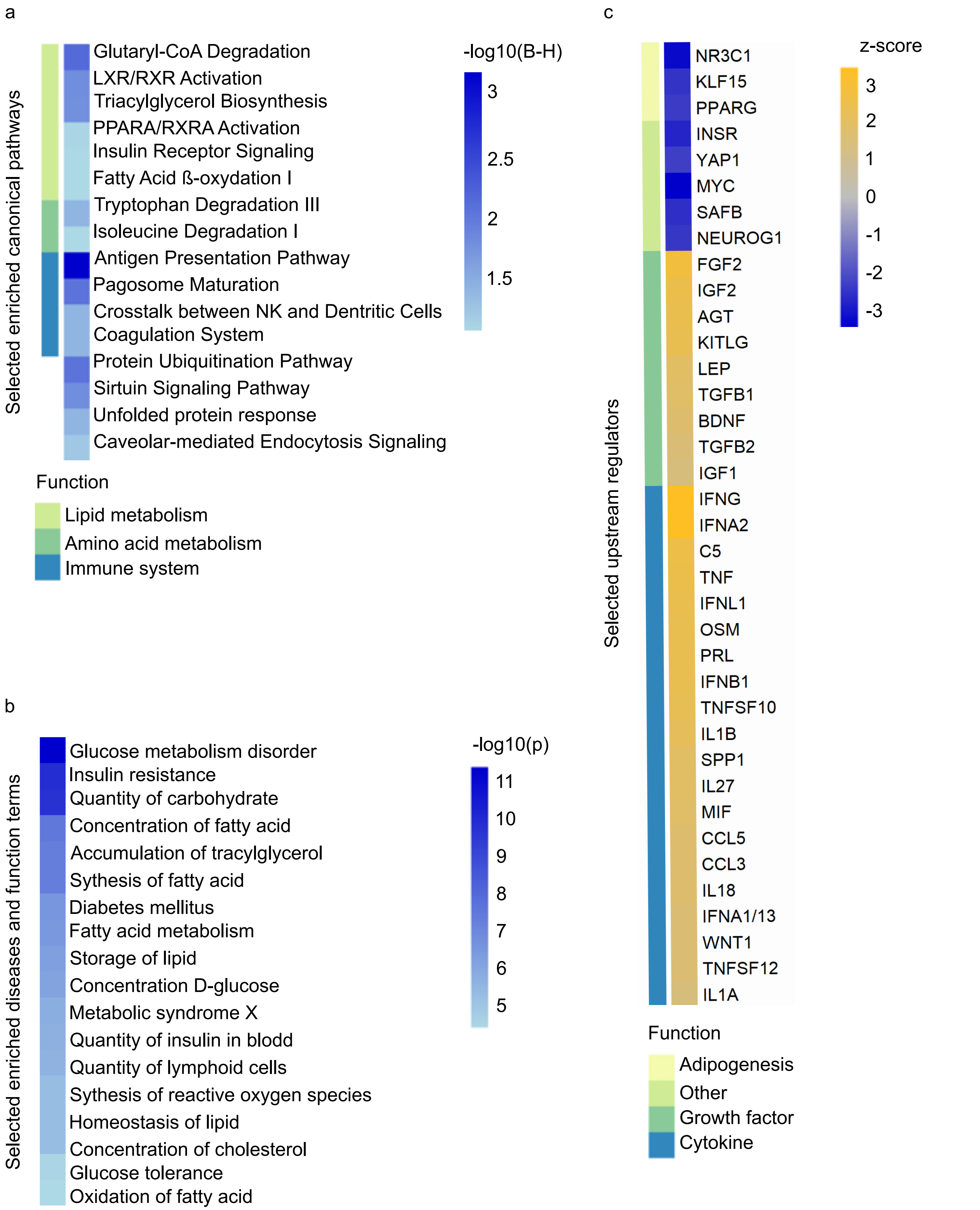
a
Selected enriched canonical pathways
Glutaryl-CoA Degradation
LXR/RXR Activation
Triacylglycerol Biosynthesis
PPARA/RXRA Activation
Insulin Receptor Signaling
Fatty Acid ß-oxydation I
Tryptophan Degradation III
Isoleucine Degradation I
Antigen Presentation Pathway
Pagosome Maturation
Crosstalk between NK and Dentritic Cells
Coagulation System
Protein Ubiquitination Pathway
Sirtuin Signaling Pathway
Unfolded protein response
Caveolar-mediated Endocytosis Signaling
-log10(B-H)
3
2.5
2
1.5
Function
Lipid metabolism
Amino acid metabolism
Immune system
b
Selected enriched diseases and function terms
Glucose metabolism disorder
Insulin resistance
Quantity of carbohydrate
Concentration of fatty acid
Accumulation of tracylglycerol
Sythesis of fatty acid
Diabetes mellitus
Fatty acid metabolism
Storage of lipid
Concentration D-glucose
Metabolic syndrome X
Quantity of insulin in blodd
Quantity of lymphoid cells
Sythesis of reactive oxygen species
Homeostasis of lipid
Concentration of cholesterol
Glucose tolerance
Oxidation of fatty acid
-log10(p)
11
10
9
8
7
6
5
c
Selected upstream regulators
NR3C1
KLF15
PPARG
INSR
YAP1
MYC
SAFB
NEUROG1
FGF2
IGF2
AGT
KITLG
LEP
TGFB1
BDNF
TGFB2
IGF1
IFNG
IFNA2
C5
TNF
IFNL1
OSM
PRL
IFNB1
TNFSF10
IL1B
SPP1
IL27
MIF
CCL5
CCL3
IL18
IFNA1/13
WNT1
TNFSF12
IL1A
z-score
3
2
1
0
-1
-2
-3
Function
Adipogenesis
Other
Growth factor
Cytokine

ESM Fig. 3 Ingenuity pathway analysis of microarray data of hMADS cells loss of function for *HAND2*. **(a, b)** Significantly regulated genes (FDR<10%) from the analysis siCtr vs si*HAND2* were used as input and enriched terms from the Canonical Pathways analysis **(a)** and Disease &Functions analysis **(b)**. **(c)** Predicted significantly activated (z-score >2) or inhibited (z-score <-2) upstream regulators. Terms and regulators were selected mainly for relevance to metabolism. All growth factors and cytokines in the upstream regulator analysis had z-scores >2 and are shown.

ESM Fig. 4

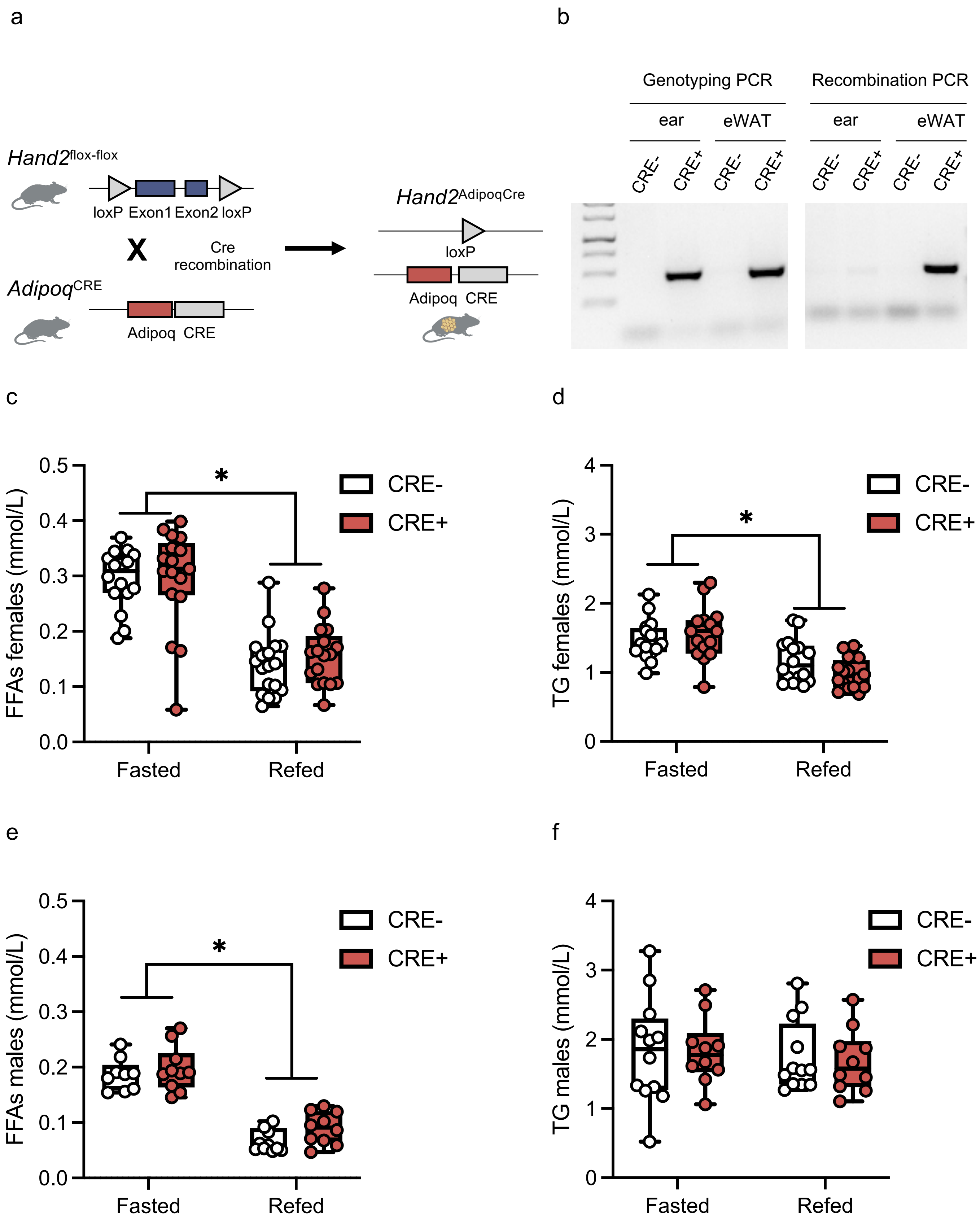
a
Hand2flox-flox
loxP Exon1 Exon2 loxP
X
Cre
recombination
AdipoqCRE
Adipoq CRE
Hand2AdipoqCre
loxP
Adipoq CRE
b
Genotyping PCR
Recombination PCR
ear
eWAT
ear
eWAT
CRE-
CRE+
CRE-
CRE+
CRE-
CRE+
CRE-
CRE+
c
FFAs females (mmol/L)
0.0
0.1
0.2
0.3
0.4
0.5
Fasted
Refed
*
CRE-
CRE+
d
TG females (mmol/L)
0
1
2
3
4
Fasted
Refed
*
CRE-
CRE+
e
FFAs males (mmol/L)
0.0
0.1
0.2
0.3
0.4
0.5
Fasted
Refed
*
CRE-
CRE+
f
TG males (mmol/L)
0
1
2
3
4
Fasted
Refed
CRE-
CRE+

ESM Fig. 4 Metabolic phenotyping of *HAND2*^{AdipoqCre} mice*.* **(a)** Breeding protocol of the *HAND2*^{AdipoqCre} mouse line. **(b)** *Hand2* DNA expression illustrating the ubiquitous expression of CRE recombinase and the adipose tissue specific recombination of *Hand2*. **(c-f)** FFA and TG levels in the serum of *HAND2*^{AdipoqCre} and wild type littermate females (n=17 mice) **(c, d)** and males (n=12 mice) **(e, f)** after 12h fasting or 6h fasting followed by 12h refeeding. Figure (**a**) was created with BioRender.com. Statistics: one-way ANOVA with Tukey test (**c-f**); median with upper and lower quartile ± maximum and minimum. Statistical significance is indicated by *$p<0.05$.

ESM Fig.5

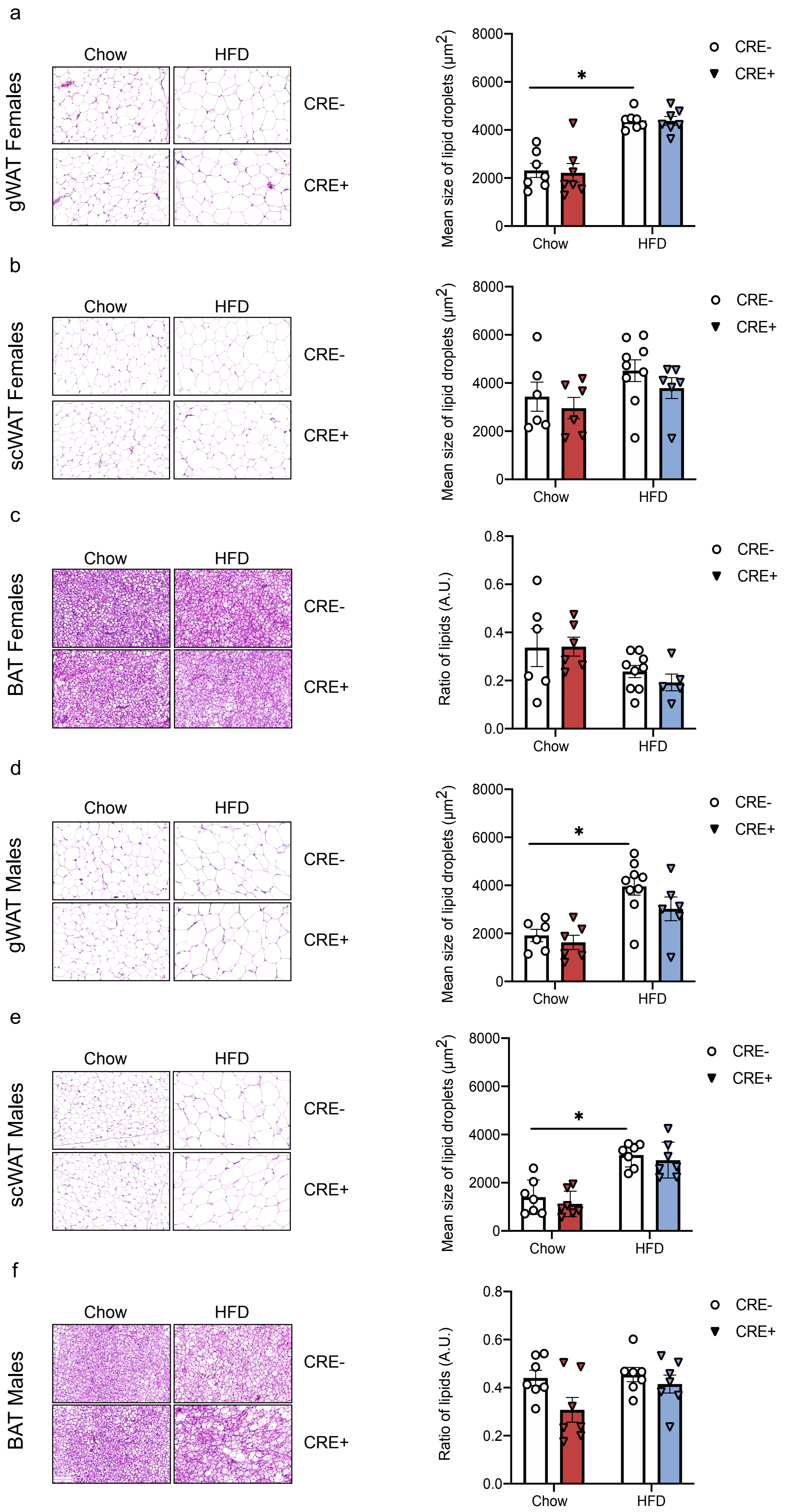

a
gWAT Females
Chow
HFD
CRE-
CRE+
Mean size of lipid droplets (µm2)
8000
6000
4000
2000
0
Chow
HFD
*
CRE-
CRE+
b
scWAT Females
Chow
HFD
CRE-
CRE+
Mean size of lipid droplets (µm2)
Chow
HFD
CRE-
CRE+
c
BAT Females
Chow
HFD
CRE-
CRE+
Ratio of lipids (A.U.)
0.8
0.6
0.4
0.2
0.0
Chow
HFD
CRE-
CRE+
d
gWAT Males
Chow
HFD
CRE-
CRE+
Mean size of lipid droplets (µm2)
Chow
HFD
*
CRE-
CRE+
e
scWAT Males
Chow
HFD
CRE-
CRE+
Mean size of lipid droplets (µm2)
Chow
HFD
*
CRE-
CRE+
f
BAT Males
Chow
HFD
CRE-
CRE+
Ratio of lipids (A.U.)
Chow
HFD
CRE-
CRE+

*

ESM Fig. 5 Histological analysis of *HAND2*^{AdipoqCre} mice fed an HFD. **(a-f)** eosin/hematoxylin staining on histological cut of the different adipose tissues of WT or *HAND2*^{AdipoqCre} mice fed a Chow or an HFD for 12 weeks. Pictures were taken and the size of the adipocytes was measured in females gWAT **(a)**, scWAT **(b)**, BAT **(c)**, and in males gWAT **(d)**, scWAT **(e)**, BAT **(f)** (n=6-9 mice). Statistics: two-way ANOVA with Tukey test; mean ± SEM. Statistical significance is indicated by *p<0.05.

ESM Fig. 6

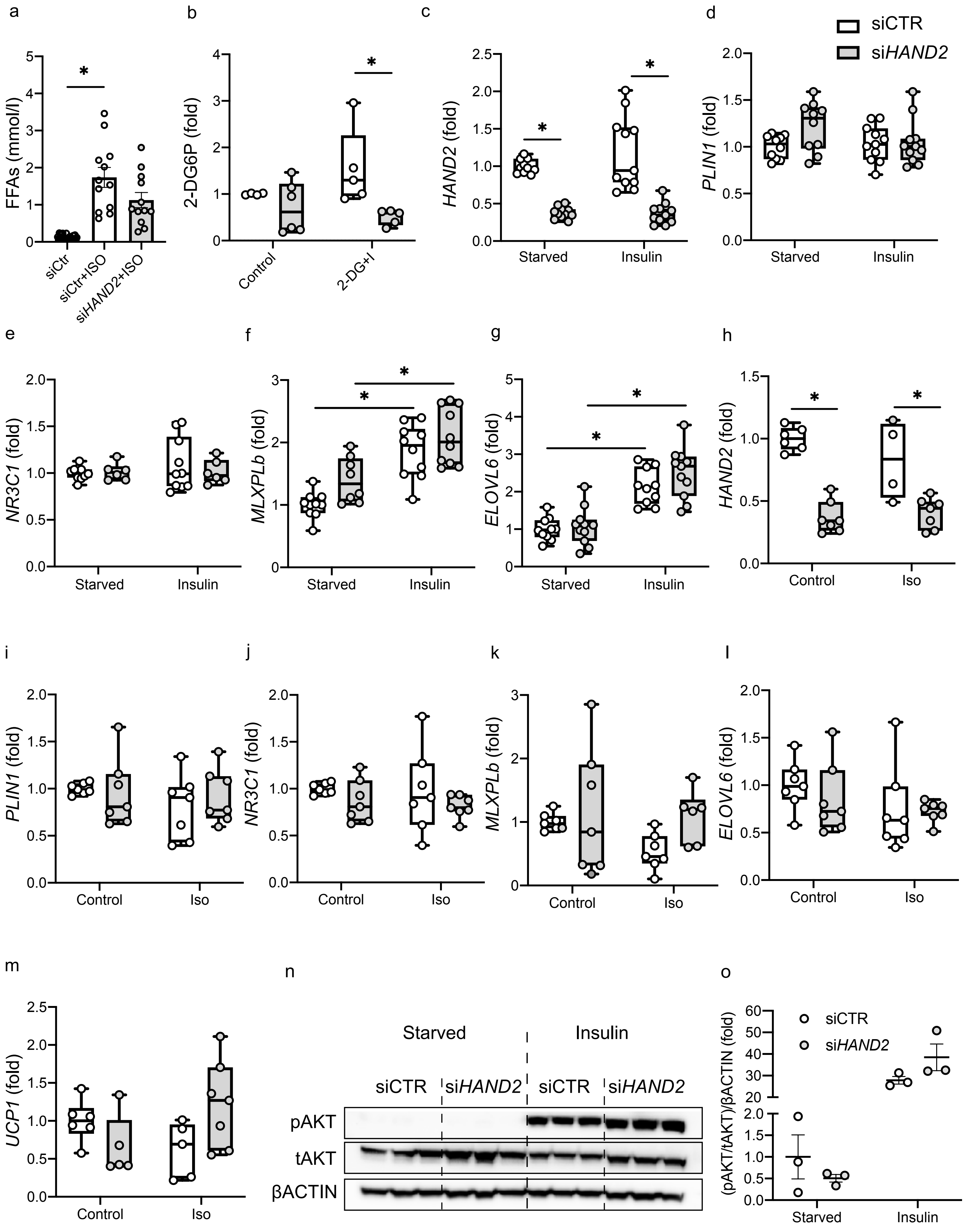

a
FFAs (mmol/l)
siCtr
siCtr+ISO
siHAND2+ISO
b
2-DG6P (fold)
Control
2-DG+I
c
HAND2 (fold)
Starved
Insulin
d
PLIN1 (fold)
siCTR
siHAND2
e
NR3C1 (fold)
f
MLXPLb (fold)
g
ELOVL6 (fold)
h
HAND2 (fold)
Control
Iso
i
PLIN1 (fold)
j
NR3C1 (fold)
k
MLXPLb (fold)
l
ELOVL6 (fold)
m
UCP1 (fold)
n
Starved
Insulin
siCTR
siHAND2
pAKT
tAKT
βACTIN
o
(pAKT/tAKT)/βACTIN (fold)

ESM Fig. 6 Phenotyping of *HAND2* loss of function in mature adipocytes in vitro. **(a, h-m)** Differentiated hMADs cell (D9) transfected with siCtr or si*HAND2* and treated 24h later with isoproterenol (10µmol/l) for 8 h. Free fatty acid concentration was measured **(a)** (n=12 replicates) as well as key gene expression **(h-m)** (n= 4-7 replicates). **(b-g)** hMADS cells were starved in insulin and with 1mol/l glucose for 12h. Glucose uptake assay was then performed **(b)** (Control n= 6 replicates, 2-DG + I n=5 replicates) or the cells were refed with 10nmol/l insulin and 10mmol/l glucose for 8h and the expression of key genes of metabolic pathways was analyzed **(c-g)** (n= 6-11 replicates). **(n, o)** hMADS cells were starved in insulin for 6h before being refed with 10nmol/l insulin for 15 minutes. The ratio between phosphorylated AKT and total AKT was measured by protein quantification from wester-blot. Data are presented as fold change compared to the condition siCtr/Starved or siCtr/Control (**a-m, o**). Statistics: one-way ANOVA with Tukey test (**a**); two-way ANOVA with Tukey test (**b-m, o**). Mean ± SEM (**a, o**); median with upper and lower quartile ± maximum and minimum (**b-m**). Statistical significance is indicated by *$p<0.05$.

ESM Fig. 7

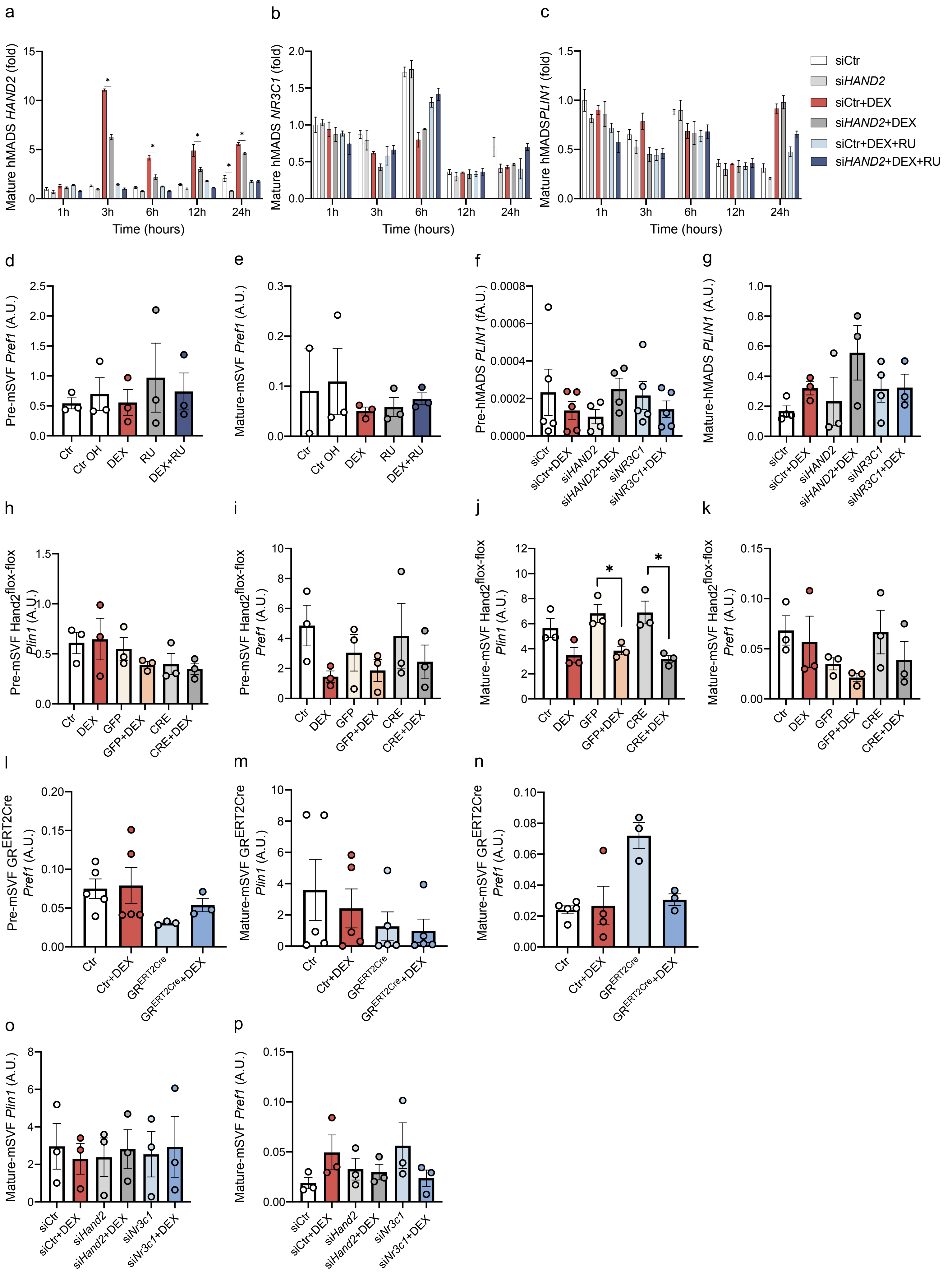

a
Mature hMADS HAND2 (fold)
Time (hours)
1h
3h
6h
12h
24h
b
Mature hMADS NR3C1 (fold)
c
Mature hMADS PLIN1 (fold)
siCtr
siHAND2
siCtr+DEX
siHAND2+DEX
siCtr+DEX+RU
siHAND2+DEX+RU
d
Pre-mSVF Pref1 (A.U.)
Ctr
Ctr OH
DEX
RU
DEX+RU
e
Mature-mSVF Pref1 (A.U.)
f
Pre-hMADS PLIN1 (fA.U.)
siCtr
siCtr+DEX
siHAND2
siHAND2+DEX
siNR3C1
siNR3C1+DEX
g
Mature-hMADS PLIN1 (A.U.)
h
Pre-mSVF Hand2flox-flox Plin1 (A.U.)
Ctr
DEX
GFP
GFP+DEX
CRE
CRE+DEX
i
Pre-mSVF Hand2flox-flox Pref1 (A.U.)
j
Mature-mSVF Hand2flox-flox Plin1 (A.U.)
k
Mature-mSVF Hand2flox-flox Pref1 (A.U.)
l
Pre-mSVF GRERT2Cre Pref1 (A.U.)
Ctr
Ctr+DEX
GRERT2Cre
GRERT2Cre+DEX
m
Mature-mSVF GRERT2Cre Plin1 (A.U.)
n
Mature-mSVF GRERT2Cre Pref1 (A.U.)
o
Mature-mSVF Plin1 (A.U.)
siCtr
siCtr+DEX
siHand2
siHand2+DEX
siNr3c1
siNr3c1+DEX
p
Mature-mSVF Pref1 (A.U.)

ESM Fig. 7 *HAND2* regulation by glucocorticoids via the glucocorticoid receptor. **(a-c)** Expression of *HAND2, NR3C1* and *PLIN1 in* differentiated hMADS cells transfected either with siCtr or si*HAND2* (20nmol/l) 24h before being treated with 1µmol/l DEX or 1µmol/l DEX + 2µmol/l RU following a kinetic (n=3 replicates). **(d, e)** *Pref1* expression in pre-mSVF **(d)** and mature mSVF **(e)** treated with 1µmol/l DEX or 1µmol/l DEX + 2µmol/l RU (n=3 replicates). **(f, g)** *PLIN1* expression in hMADS preadipocytes **(f)** and mature adipocytes **(g)** transfected with si*HAND2* or si*NR3C1* (n=4 replicates). **(h-p)** *Plin1* and *Pref1* expression in mSVF from *Hand2*$^{flox/flox}$ **(h-k)** (n=3 replicates); in mSVF from GR^{ERT2CRE} preadipocytes **(l-n)** and in mSVF from wt mice **(o, p)** (n=3 replicates). Data are presented as fold change compared to the condition siCtr/1h (**a- c**). Data are presented as arbitrary unit representing copy number normalized on *TBP* (**d-p**). Statistics: two-way ANOVA with Tukey test (**a-c**). one-way ANOVA with Tukey test (**d-p**), mean ± SEM. Statistical significance is indicated by *p<0.05.

ESM Fig. 8

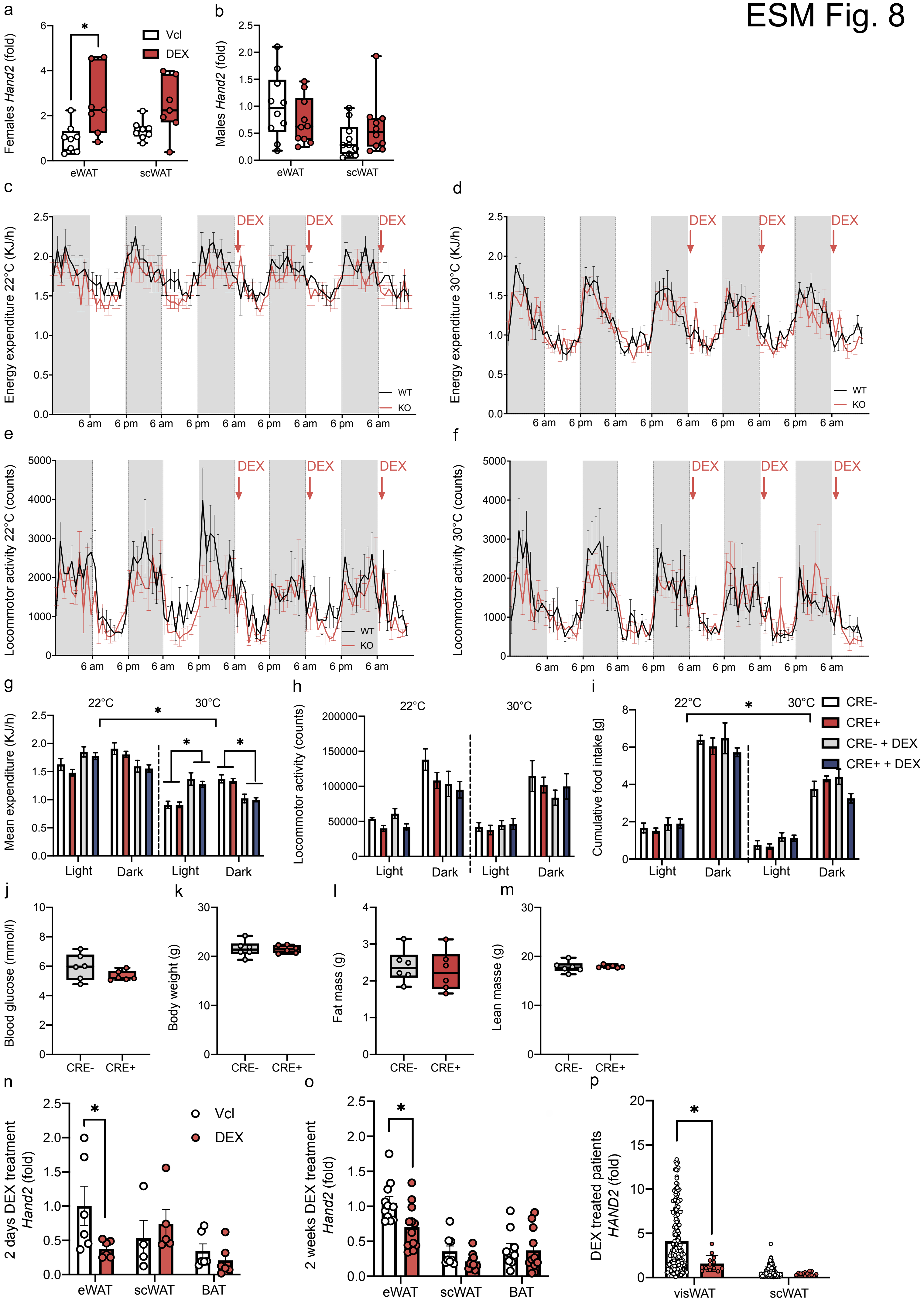
a
Females Hand2 (fold)
Vcl
DEX
eWAT
scWAT
b
Males Hand2 (fold)
eWAT
scWAT
c
Energy expenditure 22°C (KJ/h)
DEX
WT
KO
6 am
6 pm
d
Energy expenditure 30°C (KJ/h)
e
Locommotor activity 22°C (counts)
f
Locommotor activity 30°C (counts)
g
Mean expenditure (KJ/h)
22°C
30°C
Light
Dark
h
Locommotor activity (counts)
i
Cumulative food intake [g]
CRE-
CRE+
CRE- + DEX
CRE+ + DEX
j
Blood glucose (mmol/l)
k
Body weight (g)
l
Fat mass (g)
m
Lean masse (g)
n
2 days DEX treatment Hand2 (fold)
BAT
o
2 weeks DEX treatment Hand2 (fold)
p
DEX treated patients HAND2 (fold)
visWAT

ESM Fig. 8 Effect of DEX on *HAND2* expression in vivo. **(a, b)** Wild type mice were injected with DEX and scarified 6h later. *Hand2* expression was measured in gWAT and scWAT of females (Vcl n=8 mice, DEX n=7 mice) **(a)** and males (Vcl n=10 mice, DEX n=10 mice) **(b)**. **(c-m)** Females *HAND2*^{AdipoqCre} were housed in metabolic cages (CRE- n=6 mice, CRE+ n=6 mice). Energy expenditure **(c, d, g)**, activity **(e, f, h)** and food intake **(i)** were measured over a period of 5 days at 22°C or thermoneutrality (30°C), including 3 daily injections with DEX. Serum **(j)**, body weight **(k)**, fat mass **(l)** and lean mass **(m)** were recorded before the sacrifice. **(n)** *Hand2* expression in wild type animals injected daily with DEX (1mg/Kg) and scarified after 2 days (6h after the last injection) (Vcl n=6 mice, DEX n=6 mice) **(o)** and in 2 weeks treatment (Vcl n=12 mice, DEX n=12 mice). **(p)** *HAND2* expression in adipose tissue of humans receiving orally dexamethasone (1-4mg/kg per day) for more than 1 week. (Ctr n=206 individuals, DEX n=13 individuals). Data are presented as fold change compared to the condition eWAT/Vcl (**a, b, n-p**). Statistics: two-way ANOVA with Tukey test; (**a, b, g-l, n-p**); two-tailed unpaired t-test (**j-m**). median with upper and lower quartile ± maximum and minimum (**a, b, j-m**), mean ± SEM (**c-i, n-p**). Statistical significance is indicated by *$p<0.05$.

ESM Fig. 9

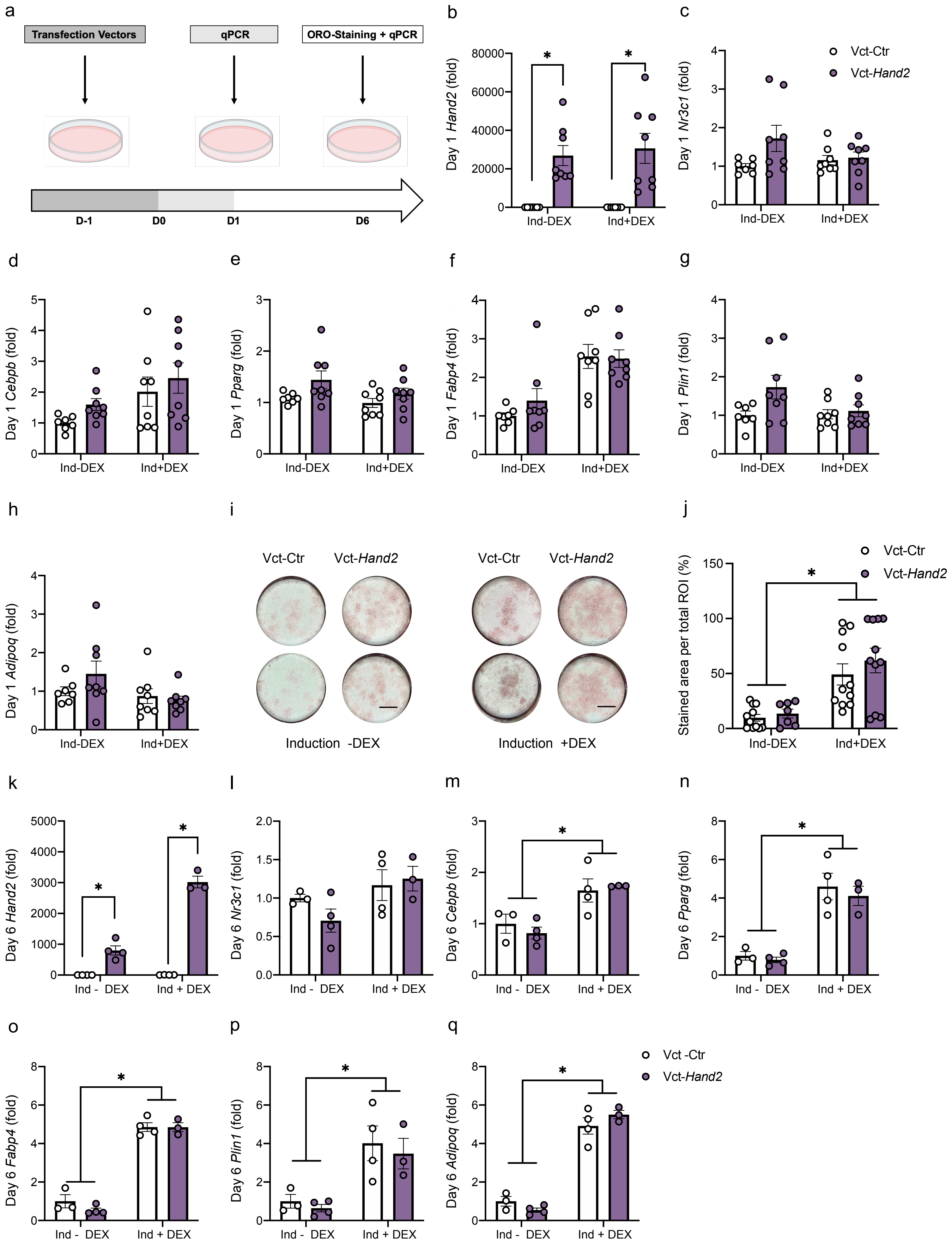

a
Transfection Vectors
qPCR
ORO-Staining + qPCR
D-1
D0
D1
D6
b
Day 1 Hand2 (fold)
Ind-DEX
Ind+DEX
c
Day 1 Nr3c1 (fold)
Vct-Ctr
Vct-Hand2
d
Day 1 Cebpb (fold)
e
Day 1 Pparg (fold)
f
Day 1 Fabp4 (fold)
g
Day 1 Plin1 (fold)
h
Day 1 Adipoq (fold)
i
Vct-Ctr
Vct-Hand2
Induction -DEX
Induction +DEX
j
Stained area per total ROI (%)
k
Day 6 Hand2 (fold)
Ind - DEX
Ind + DEX
l
Day 6 Nr3c1 (fold)
m
Day 6 Cebpb (fold)
n
Day 6 Pparg (fold)
o
Day 6 Fabp4 (fold)
p
Day 6 Plin1 (fold)
q
Day 6 Adipoq (fold)
Vct -Ctr
Vct-Hand2

ESM Fig. 9 *Hand2* expression is required for differentiation but does not compensate GR stimulation. **(a)** Experimental protocol of Hand2 over-expression in 3T3L1. **(b-h)** 3T3L1 cells at day 1, 48h after transfection and 24h after induction of the differentiation. Expression of *Hand2, Nr3c1, Cebpb, Pparg, Fabp4, Plin1* and *Adipoq* in (n=7-8 replicates). **(i, q)** 3T3L1 cells at day 6, transfected a day -1. Oil redo staining **(i)** and quantification **(j)** (n=11 replicates). Expression of *Hand2, Nr3c1, Cebpb, Pparg, Fabp4, Plin1* and *Adipoq* **(k-q)** (n=3-4 replicates). Data are presented as fold change compared to the condition Ind-DEX/Vct-Ctr (**b-h, k-q**). Statistics: two-way ANOVA with Tukey test; mean ± SEM (**b-h, j-q**). Statistical significance is indicated by *p<0.05. Ind, Induction of differentiation.

ESM Fig. 10

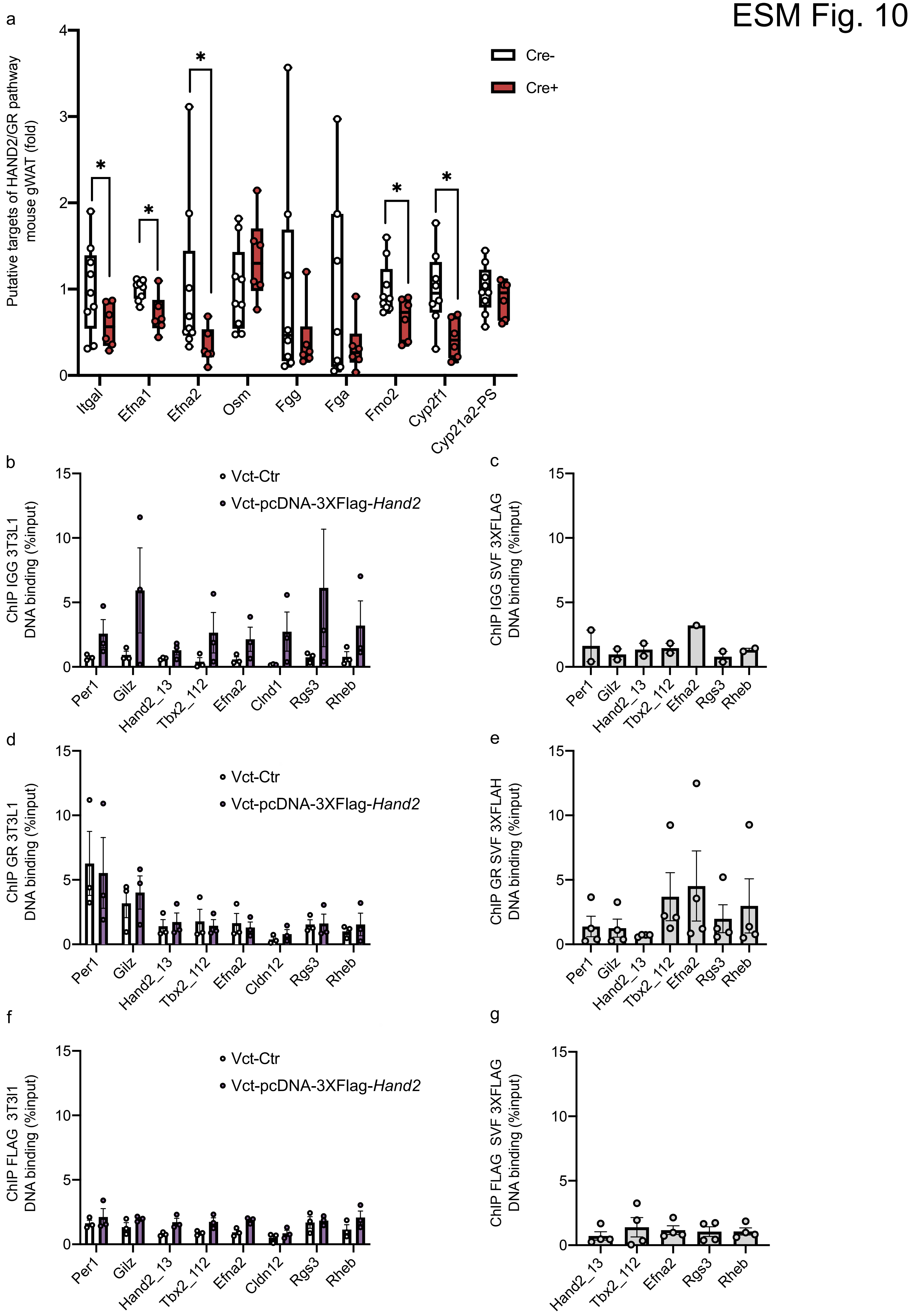
a
Putative targets of HAND2/GR pathway mouse gWAT (fold)
Cre-
Cre+
*
Itgal
Efna1
Efna2
Osm
Fgg
Fga
Fmo2
Cyp2f1
Cyp21a2-PS
b
ChIP IGG 3T3L1 DNA binding (%input)
Vct-Ctr
Vct-pcDNA-3XFlag-Hand2
Per1
Gilz
Hand2_13
Tbx2_112
Efna2
Clnd1
Rgs3
Rheb
c
ChIP IGG SVF 3XFLAG DNA binding (%input)
Per1
Gilz
Hand2_13
Tbx2_112
Efna2
Rgs3
Rheb
d
ChIP GR 3T3L1 DNA binding (%input)
Vct-Ctr
Vct-pcDNA-3XFlag-Hand2
Per1
Gilz
Hand2_13
Tbx2_112
Efna2
Cldn12
Rgs3
Rheb
e
ChIP GR SVF 3XFLAH DNA binding (%input)
Per1
Gilz
Hand2_13
Tbx2_112
Efna2
Rgs3
Rheb
f
ChIP FLAG 3T3l1 DNA binding (%input)
Vct-Ctr
Vct-pcDNA-3XFlag-Hand2
Per1
Gilz
Hand2_13
Tbx2_112
Efna2
Cldn12
Rgs3
Rheb
g
ChIP FLAG SVF 3XFLAG DNA binding (%input)
Hand2_13
Tbx2_112
Efna2
Rgs3
Rheb

ESM Fig. 10 Confirmation of HAND2 targets. **(a)** Expression of HAND2 putative targets in gWAT of male *HAND2*^{AdipoqCr} mice (Cre- n=9 mice, Cre+ n=6 mice). **(b-g)** ChIP-qPCR analysis of selected GR or HAND2 binding sites was performed on 3T3L1 transfected with pcDNA-3XFlag-*Hand2* vector (n=3 replicates) **(b, d, f)** and scWAT SVF from *Hand2*^{3XFlag} mice (n=2-4 replicates) **(c, e, g)**. ChIP-qPCR was used to amplify chromatin derived from immunoprecipitations with anti-IgG antibody, used as control **(b, c)**, anti-GR antibody **(d, e)** and anti-Flag antibody **(f, g)**. Data are presented as fold change compared to the condition CRE- (**a**). Statistics: two-way ANOVA with Tukey test (**a, b, d, f**), one-way ANOVA with Tukey test (**c, e, g**); median with upper and lower quartile ± maximum and minimum (**a**), mean ± SEM (**b-g**). Statistical significance is indicated by *$p<0.05$.
